# Assessing the quality of whole slide images in cytology from nuclei features

**DOI:** 10.1016/j.jpi.2025.100420

**Published:** 2025-02-11

**Authors:** Paul Barthe, Romain Brixtel, Yann Caillot, Benoît Lemoine, Arnaud Renouf, Vianney Thurotte, Ouarda Beniken, Sébastien Bougleux, Olivier Lézoray

**Affiliations:** a Datexim, Caen 14000, France; b Normandie Univ, UNICAEN, ENSICAEN, CNRS, GREYC, Caen 14000, France

**Keywords:** Automated quality control, Digital pathology, Cytology, Whole slide image analysis, Out-of-distribution detection

## Abstract

**Background and objective:**

Implementation of machine learning and artificial intelligence algorithms into digital pathology laboratories faces several challenges, notably the variation in whole slide image preparation protocols. The diversity of preparation pipelines forces algorithms to be protocol-dependant. Moreover, the error susceptibility of each stage in the preparation process implies a need of quality control tools. To address these challenges, this article introduces a straightforward, interpretable, and computationally efficient quality control module to ensure optimal algorithmic performance.

**Methods:**

The proposed quality control module ensures algorithmic performance by representing an algorithm by a reference whole slide image preparation protocol validated on it. Then, inspired by data description methods, a preparation protocol is represented by nuclei feature distributions, obtained for several whole slide images it has produced. The quality of a preparation protocol is evaluated according to several reference preparation protocols, by comparing their feature distributions with a weighted distance.

**Results:**

Through empirical analysis conducted on seven distinct preparation protocols, we demonstrated that the proposed method build a quality module that clearly discriminates each preparation. Additionally, we showed that this module performs well on more larger and realistic corpus from laboratories routine, detecting quality deviations.

**Conclusion:**

Even if the proposed method necessitates minimal data and few computational resources, we showed that it is interpretable and relevant on realistic corpus from several laboratories' routine. We strongly believe in the necessity of quality control from the algorithmic perspective and hope this kind of approach will be extended to improve quality and reliability of digital pathology whole slide images.

## Introduction

The Digital Pathology Association defines digital pathology (DP) as encompassing tools and systems for digitizing pathology slides and associated metadata, including their storage, review, and analysis, as well as the enabling infrastructure.^1^ WSIs are the numerical representation of glass slides and are often very large files, sometimes exceeding a gigabyte, which poses challenges for storage and transfer. This is why, in recent decades, advances in computational technology and storage have provided opportunities for DP to grow quickly. Computer-aided diagnosis (CAD) systems play a crucial role in the review and analysis of whole slide images (WSIs), aiming to enhance diagnostic accuracy and efficiency of pathology experts by using the computer output as a guide. Such systems and, in turn, DP laboratories, have recently benefited from advances in artificial intelligence (AI) and machine learning (ML). For that reason, DP is now sometimes used as a more “generic term that includes AI-based approaches for detection, segmentation, diagnosis, and analysis of WSIs.”.^34^ Despite these advancements, the implementation of CAD systems within a WSI preparation pipeline (WSI-PP) is hindered by the diversity of preparation protocols used. Different protocols result in WSIs with various characteristics, necessitating the customization of CAD algorithms to match these unique features. Before using an algorithm, laboratories must then check that their WSI-PPs meet the algorithm's requirements, in order to achieve optimal algorithmic performance. This article specifically addresses such needs of quality control (QC) for cytological laboratories.

### Digital cytology laboratory workflow

Digital cytology is a branch of DP focusing on the analysis of cells in biological samples. To better understand why cytological WSIs are prone to quality errors, we briefly describe the four main stages of a digital cytology laboratory workflow: sample collection, sample preparation, sample coloration, and slide digitization. As described by Mach et al.,^31^ sample collection includes phlebotomy, fluid aspiration, or saline washing of a mucosal surface with catheter assistance. Once the sample is collected, an aliquot of this biofluid is centrifuged or filtered, usually with an automated instrument, to collect cell sediments and evenly distribute them on a glass slide. Subsequently, automated systems stained the slide with specific dyes and either alcohol-fixed or air-dried to remove water content before the glass cover strip is applied. Finally, scanners are used to digitized the glass slide into a WSIs.

### WSI analysis

**Fig. 1:**
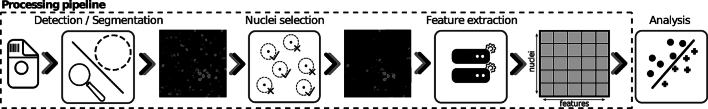
Overview of a typical cytological WSI analysis workflow. The analysis module icon represents an analysis with a classification objective.

As cytology involves cell analysis, typically via the nucleus, we focus here on the WSI analysis workflow from a nuclei point of view. This classical workflow^31^ can be divided into four stages, as depicted in Fig. 1. First, nuclei detection is performed on the WSI to identify areas in which the nuclei are located. Following this, fine-grained information about detected object boundaries is provided via segmentation. Then, segmented objects can be filtered by any heuristic and features are calculated and assigned to each resulting object. The analysis of these segmented objects is then conducted by a processing module, which may utilize various approaches (e.g., expert systems or machine learning) and pursue different objectives (e.g., transformation, feature analysis, or classification). It should be noted that henceforth in this article, the term nuclei will refer to segmented objects. However, it is known that a segmented object equates to a nucleus only if the object detection and segmentation are flawless.

### Quality control

As mentioned above, the analysis of WSIs can only achieve optimal algorithmic performance if the WSI-PP meets the algorithm's requirements. However, each stage of the WSI-PP can affect the quality of the WSI. For sample collection, depending on the technique and the surgeon, the sample can vary in appearance, and artifacts may be introduced. For sample preparation, each automated system manufacturer has its own settings and operational methods, which produce staining with different characteristics. For slide digitization, numerous WSI scanners are available on the market, each with its own hardware and software components, leading to significant variations in WSIs characteristics. However, as highlighted by Janowczyk et al.,^18^ manual review of glass and digital slides is laborious, qualitative, and subject to intra- and inter-reader variability, underscoring the need for an automated and reproducible method to assess WSI quality. In response to these challenges, we propose a straightforward, interpretable and computationally efficient QC module, to assess WSI-PP quality with respect to (w.r.t.) reference WSI-PPs. This module is capable of raising quality alerts during its creation, prompting a reconsideration of reference WSI-PPs. Once created, the module measures the similarity between reference preparation pipelines, representing algorithms, and the tested preparation pipeline. If the used measure is not satisfactory, an alert is raised.

In next section, we explore and show limitations of existing research, highlighting the need for further advancements. To address this gap, third section details the method pursued to build our QC module and introduce the material used to: empirically determine optimal parameter values for the QC module and confront this module to real-world production environments. Finally, we present experiments and discuss results of our QC module on real-world production environments.

## Related works

WSI quality is highly dependent on the WSI-PP. Each stage can introduce severe artifacts (unexpected objects), prejudicial for human expert interpretation and representing potential algorithm failure cases. Identifying artifacts in a pre-processing step is then required. We review QC pipelines created to handle artifacts from preparation and digitization. Then, we introduce QC methods that have been designed for WSI-PP and highlight the fact that QC from an algorithm admissibility perspective is poorly represented in the DP literature. We then discuss about the importance of such QC and present works dealing with the out-of-distribution (OOD) problem, which are more akin to our proposed method.

### Quality control modules

The importance of implementing QC is illustrated in Song et al.,^46^ where they trained a deep network on histopathological WSIs digitized with a specific scanner. Then, they digitized a WSI test set with three scanner models, including the scanner used during the training stage, and inferred the network on this set. They observed a significant difference in performances between the scanner used to create the training set and the two other scanners. Moreover, Ogura et al.^35^ showed that even the same scanner instance on the same WSI can produce different results at different times. It is in response to these concerns that QC modules have been built.

A QC module, based on sharpness features extracted from cells, is proposed by Lahrmanb et al.^23^ A WSI sharpness score is used jointly with a user-defined threshold in order to consider a WSI as analyzable. In Ameisen et al.,^2^ a WSI is considered for further use according to scores evaluating tile blurriness, contrast, brightness, and color at several magnification levels. Campanella et al.^5^ used sharpness measures from patches jointly with a random forest classifier to provide a blur slide-level score. Another slide-level score, is introduced by Zhang et al.,^52^ with the use of a CNN. Shakhawat et al.^41^ present a quality evaluation method which distinguishes two origins of quality degradation, preparation or digitization. They used a support vector machine (SVM) classifier to detect air bubbles and tissue folds artifacts in histological WSIs in order to inform on slide preparation quality. For digitization, only WSI regions without artifacts are analyzed. Then, based on Hashimoto et al.,^16^ sharpness and noise features are computed on artifact-free zones and fed to a linear regression model to evaluate slide quality. Additionally to the development of a multi-class deep learning model for semantic segmentation artifacts, Smit et al.^43^ proposed a QC module that informs on the actions that must be done to fix artifacts' issues. In Haghighat et al.,^13^^,^^14^ a WSI is accepted or rejected according to a slide-level quality score based on predicted patch-level quality measures from a multi-label deep neural network.

We present in the following a framework, designed for QC modules, and place these studies into it in order to highlight the fact that a specific QC is not represented.

### Integrating quality control in digital pathology

Given the importance of QC for preparation pipelines, several studies have examined the limitations of AI applied to DP.^3^^,^^8^^,^^39^ They discussed how advances in AI should be considered and the challenges that need to be overcome before the adoption of AI, in particular for ML models.

**Fig. 2:**
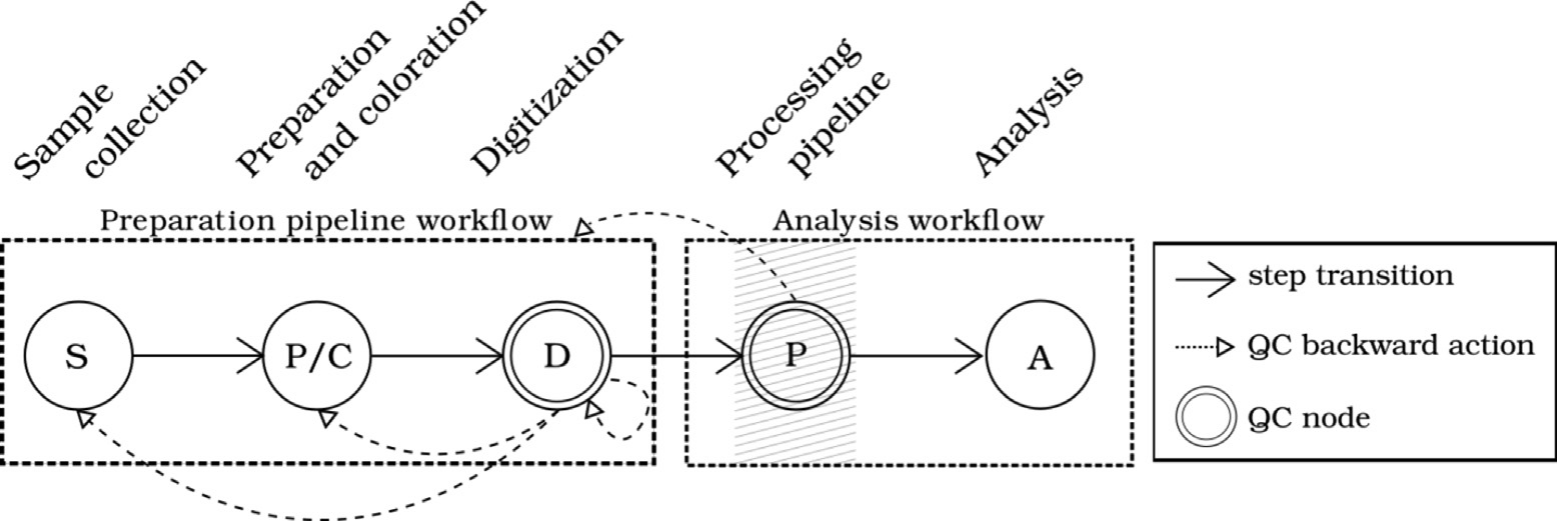
Prototypical QC module framework. Modified version of Fig. 13 in Brixtel et al.^4^ The ‘Analysis workflow’ refers to Fig. 1. Hatched area indicates where QC is performed in the present work.

QC modules have also been designed, like in Smith et al.,^44^ where they discussed about issues in WSIs' analysis before dawning up a QC module framework for deep learning project. More generically, Brixtel et al.^4^ suggested integrating QC into every stage of the DP diagnosis pipeline, and proposed a QC module framework. Fig. 2 is taken from their article and slightly modified it to suit our needs. In this framework, two QC checks are performed on two different nodes, one after slide digitization and one before the analysis. The first node ensures that the WSI meets the requirements w.r.t. human interpretation and artifacts presented until then (e.g., bubbles, foreign objects, blood, or out-of-focus effect). The second node ensures that the analysis module can be used as intended by being aware of its range of acceptability. It checks that the objects taken as input are coherent w.r.t. what the analysis module has been designed for. For example, a ML algorithm can be self-aware of its range of acceptability by analyzing its training data and by comparing inputs with this range.

All the studies cited for quality control are based on the human perspective to perform QC and answer the question “Can this WSI be analyzed by a pathology expert?”. In other words, they are only addressed to the QC digitization module (digitization node of Fig. 2). None of them are addressed to the QC analysis module (analysis node of Fig. 2): assessing a good WSI digitization quality from human perspective does not necessarily imply a good WSI digitization quality for analysis.

### Limitation of human-based quality assessments in digital pathology

Humans cannot detect all the noise distortions that might interfere with an algorithm performance. Therefore, evaluating WSI digitization quality from a human perspective does not necessarily ensure good quality for automated analysis. To exhibit this, Ullman et al.^50^ introduced the concept of minimal recognizable configurations of images (MIRCs), which are the smallest image crops for which human observers can still predict the correct class. After training deep neural networks on the full images, they tested them on this MIRC dataset and showed that human performance clearly outperformed deep networks. This underlines the fact that modified images may not interfere with human perspective, but with automated analysis. Concept like one-pixel-attack^47^ exhibits this by modifying the value of one pixel in order to completely change the results of an AI algorithm. Even if preventing such an attack is clearly not the main objective of a QC module, it highlights the fact that human and algorithm interpretation of a WSI are clearly not similar. Although the majority of one pixel attacks are against deep learning frameworks, Chen et al.^7^ developed and tested a gradient-based attack against a SVM model working directly in the pixel domain. Applied to WSI, Korpihalkola et al.^21^^,^^22^ show that there exists minor one-pixel modification methods that can reverse the automatic diagnosis result of a WSI.

We propose in this article a QC module to assess cytology WSI quality from the algorithm point of view. Assessing QC from an algorithm perspective is already studied in various areas, which we present in the next section.

### Algorithm-centric quality control approaches

Assessing QC from an algorithm perspective is done by preventing a model to process samples that cannot be managed. To do this, works have been done to detect OOD samples before or after applying the algorithm.

In a first approach, outliers are detected by analyzing the confidence in the model's predictions. As deep neural networks tend to produce overconfident predictions,^12^ works have been done to separate the confidence scores between in- and out-of-distribution samples. A baseline, based on probabilities from softmax distributions, is introduced by Hendrycks and Gimpel.^17^ More effective approaches are then proposed, as in Liang et al.,^27^ where they use temperature scaling jointly with small perturbations on the input data to better separate the softmax score distributions. Some other OOD methods train a specific deep model to provide, in addition to the prediction, an uncertainty score, using Bayesian neural networks,^11^ deep ensembles,^24^ or multi-head convolutional neural networks.^30^

A second approach is to use data description methods to identify OOD samples. Data description methods make a description of a training set of samples in order to detect if a new sample resemble to this training set. Tax and Duin^49^ proposed a method, inspired by the SVM paradigm, to obtain a spherically shaped boundary with minimum volume around the complete training samples. As performances of such SVM methods are suboptimal on complex and high dimensional datasets, Chalapathy et al.^6^ derived the SVM loss function for a one-class neural network model with the objective of creating a tight envelope around normal data. Lee et al.^25^ proposed that pre-trained features can be effectively modeled using a class-conditional Gaussian distribution. Using this model, they developed a confidence score to determine how closely a new sample aligns with the fitted distributions.

Similar OOD detection methods have been considered for DP datasets from different DP fields, e.g., in histology^28^^,^^29^, radiology,^33^ sonography,^20^ and ophthalmology.^26^ To the best of our knowledge, no similar work has yet been done for cytology, which we propose in the following sections.

## Materials and methods

We propose to control the quality of WSI-PPs from the features extracted by the processing pipeline just before analysis (hatched area in Fig. 2). As described in Fig. 1, we assume that the processing pipeline extracts nuclei features. Inspired by data description methods, a WSI-PP is represented by feature distributions, one per nucleus feature, obtained for several WSIs it has produced. The quality of a WSI-PP is evaluated according to several reference WSI-PPs, by comparing their feature distributions with a weighted distance. The weights and the other parameters of the proposed QC method are determined according to a validation process based on grid search. The resulting QC module can trigger quality alerts during both test and validation.

### WSI-PP representation from the processing pipeline

This section describes the feature extraction steps, and their parameters, used to represent a WSI-PP by nuclei feature distributions from a batch of $n_{\mathrm{wsi}}$ WSIs it has produced.

#### Nuclei feature extraction

We assume that the processing pipeline is composed of two main blocks: nuclei detection and segmentation (denoted $\phi_{\mathrm{nuc}}$), and nuclei feature extraction (denoted $\phi_{\text{feat}})$. Given a WSI, or more generally a batch S of $n_{\mathrm{wsi}}$ WSIs, $\phi_{\mathrm{nuc}}$ extracts a total of $n_{\mathrm{nuc}}$ nuclei, i.e., $\frac{n_{\mathrm{nuc}}}{n_{\mathrm{wsi}}}$ per WSI, and $\phi_{\text{feat}}$ extracts $n_{\text{feat}}$ scalar features per extracted nucleus. We refer to the application of the two blocks as $\phi \left(S\right)≔\phi_{\text{feat}}\left(\phi_{\mathrm{nuc}}\left(S\right)\right)$. In order to simplify the notations, the output nuclei features are encoded by a $n_{\mathrm{nuc}}\times n_{\text{feat}}$ feature matrix $C=\phi \left(S\right)$. The number and nature of the nuclei features we used in the experiments are described later. In this article, for any matrix A, its elements are denoted $A_{i,j}$ and its columns $a_{j}$, i.e., $A={\left(A_{i,j}\right)}_{i,j}={\left[a_{j}\right]}_{j}$.

#### Nuclei selection

In order to extract representative nuclei, $\phi_{\mathrm{nuc}}$ is parameterized by a composition of nuclei selection methods, and by the number $n_{\mathrm{nuc}}$ of nuclei to be selected. Three base nuclei selection methods were considered in the experiments:

• The first one, denoted $\phi_{\mathrm{nuc}}^{\text{intern},\tau }$, avoids selecting nuclei located in regions with high nucleus density (clusters). Indeed, the high rate of overlaps between nuclei can lead to segmentation problems. Due to the centrifugation process, clusters are often present at the WSI edges, nuclei are only extracted inside an ellipse centered at the WSI center. The axes of the ellipse are defined as a percentage τ of the WSI width and height. The values τ=20%,40% were tested in the experiments.
• The second method, denoted $\phi_{\mathrm{nuc}}^{\text{distrib}}$, avoids oversampling nuclei in clusters. It extracts evenly distributed nuclei from the WSI.
• The last method, denoted $\phi_{\mathrm{nuc}}^{\text{debris}}$, ignores nuclei that are labeled as debris. These preparation artifacts can distort the expressiveness of features and introduce bias. The elements on which a nucleus is considered as debris are exposed in the experiments section.

These base selection methods can be combined, together with the number of extracted nuclei. Several configurations were opposed in the experiments.

#### Reference WSI-PPs and nuclei feature selection

Some of the nuclei features considered by $\phi_{\text{feat}}$ may be correlated or more relevant in representing WSIs produced by a specific WSI-PP, for both QC and analysis. Moreover, by eliminating the less relevant features, the interpretability and the demand for computing resources should be improved. To this end, we assume to know $n_{\mathrm{ref}}$ reference WSI-PPs, with different preparation characteristics. The processing pipeline can be adapted to a specific WSI-PP i by reducing the number of nuclei features to $n_{\mathrm{sel}}\leq n_{\text{feat}}$. The reduction is encoded by a $n_{\text{feat}}\times n_{\mathrm{sel}}$ reduction matrix $P^{i}$ adapted to the WSI-PP i, i.e., an assignment from $n_{\text{feat}}$ features to $n_{\mathrm{sel}}$ features: $P_{l,r}^{i}\in \left\{01\right\}$ for all (*l*, *r*), $\sum_{l}P_{l,r}^{i}=1$ for all *r*, and $\sum_{r}P_{l,r}^{i}\leq 1$ for all *l*. The reduction matrices are defined by feature selection from the nuclei feature values obtained for all references. Then, given a batch S of WSIs and a reference WSI-PP *i*, the nuclei feature matrix adapted to reference *i* is given by $C=\phi \left(S\right)\cdot P^{i}$_._

### Nuclei feature distributions

In the QC module, a WSI-PP is represented by nuclei feature probability distributions, one per nuclei feature. Given a nuclei feature matrix $C\in {\mathbb{R}}^{n_{\mathrm{nuc}}\times n_{f}}$ with $n_{f}$ nucleus features (reduced or not), it is mapped to a matrix $F=f\left(C\right)\in {\left[01\right]}^{n_{\mathrm{bin}}\times n_{f}}$, where each column $f_{l}\in {\left[01\right]}^{n_{\mathrm{bin}}}$ is a normalized histogram with $n_{\mathrm{bin}}$ bins computed by f:

(1) $$f\left(C\right):\left\{\begin{array}{cc} \forall l, & {\tilde{c}}_{l}=\frac{c_{l}-m_{l}^{-}}{m_{l}^{+}-m_{l}^{-}}\\ \forall l, & h_{l}={\mathrm{hist}}_{\left[0,1\right]}\left({\tilde{c}}_{l}\right)\\ & s=\sum_{l}1^{⊤}\cdot h_{l}\\ \forall l, & f_{l}=\frac{h_{l}}{s} \end{array}\right)$$

For each feature *l*, the first step normalizes the input feature values to [0,1] w.r.t. reference minimum and maximum feature values $m_{l}^{-}$ and $m_{l}^{+}$, respectively, fixed later. The second step computes a histogram from the normalized values, obtained by discretizing [0,1] with $n_{\mathrm{bin}}$ equal-width bins. The last step ensures that the bin values sum to 1 (discrete probability distribution). We do not assume that all input feature values are in the range $\left[m_{l}^{-},m_{l}^{+}\right]$, i.e., some normalized values may be outside [0,1]. These eventual out-of-distribution values (${\tilde{C}}_{k,l}\notin \left[01\right]$) are not counted by the histogram function, but implicitly taken into account by the last normalization. Also, note that $f\left(C\cdot P\right)=f\left(C\right)\cdot P$ holds for any reduction matrix P.

### Quality control and measure

This section describes the QC process. It is based on the knowledge of $n_{\mathrm{ref}}$ reference WSI-PPs. Each reference WSI-PP *i* is represented by a tuple $\left(R^{i},P^{i},\alpha_{i}\right)$ from a reference batch $S^{i}$ of $n_{\mathrm{wsi}}$ WSIs produced by *i*, where: $R^{i}=f\left(\phi \left(S^{i}\right)\right)\cdot P^{i}$ is the nucleus feature distribution matrix adapted to *i* via the reduction matrix $P^{i}$. The weight $\alpha_{i}\in \left[01\right]$ represents the distance value at which preparation pipelines are considered dissimilar to the reference *i*, w.r.t. a distance function between feature distributions.

### Distance between feature distributions

**Fig. 3:**
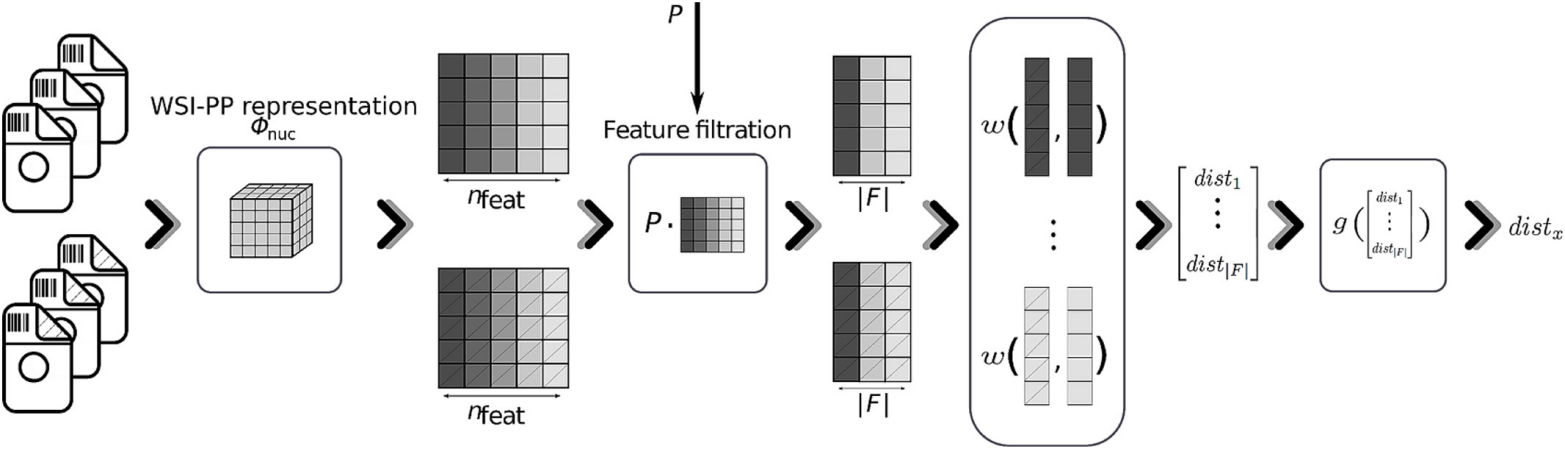
Overview of the dissimilarity measure between two WSI-PPs, w.r.t. to the selection matrix *P*. WSI-PP representations are obtained via sets of WSIs. These pipelines are then represented via a subset of features depicted by the selection matrix *P*. For each of these features, the distance function *w* is performed. Final dissimilarity distance is obtained after analyzing these distances by the aggregator function *g*.

In the QC module, WSI-PPs are compared w.r.t. their nuclei feature distributions. Let $w:{\left[01\right]}^{n_{\mathrm{bin}}}\times {\left[01\right]}^{n_{\mathrm{bin}}}\to \left[01\right]$ be a distance function between two probability distributions. We define the distance between two distribution matrices $F\in {\left[01\right]}^{n_{\mathrm{bin}}\times n_{f}}$ and $F^{\prime }\in {\left[01\right]}^{n_{\mathrm{bin}}\times n_{f}}$ by aggregating the distances obtained by ω for each feature:

(2) $$d(F,F^{'})≔g\left(\left\{\omega \left(f_{l},f_{l}^{'}\right),l=1,\ldots ,n_{f}\right\}\right),$$

where *g* aggregates values of the input set, see Fig. 3. We assume that $d\left(\cdot ,\cdot \right)\in \left[01\right]$. We considered in the experiments:

• For ω, the Wasserstein,^1^ ^19^ the Bhattacharyya^40^ and the Jensen-Shannon^2^ ^10^ distances, denoted $\omega_{\text{wass}}$, $\omega_{\text{bhat}}$, and $\omega_{\text{jsha}}$, respectively.
• For *g*, the max, the mean or the median operators, refereed as $g_{\max}$, $g_{\text{mean}}$, and $g_{\mathrm{med}}$.

#### Quality constraints

In order to control the quality of an input preparation pipeline S, we define S as compatible with a reference *i* if its nuclei feature distributions are similar to the reference distributions $R^{i}$ and dissimilar to the other reference distributions $R^{j}$, w.r.t. the nuclei features of each reference. For each reference $j=1,\ldots ,n_{\mathrm{ref}}$, the notion of (dis)similarity is controlled by the weight $\alpha_{j}\in \left(01\right)$:

(3) $$d\left(R^{i},f\left(\phi \left(S\right)\right)\cdot P^{i}\right)<\alpha_{i},$$

(4) $$\forall j\neq i,\;d\left(R^{j},f\left(\phi \left(S\right)\right)\cdot P^{j}\right)\geq \alpha_{j}.$$

These $n_{\mathrm{ref}}$ constraints can be used to control the quality of a preparation pipeline that should be compatible with a reference preparation pipeline *i* (prior knowledge), i.e., the preparation should be reconsidered if all constraints are not met, or at least the quality should be examined.

#### Quality control process

Given an input batch S of WSIs and a reference WSI-PP *i*, it is encoded by the distance vector $d={\left(d_{j}(S)\right)}_{j=1,\ldots ,n_{\text{ref}}}$ to all $n_{\mathrm{ref}}$ references, including *i*, where

(5) $$\forall j,\;d_{j}\left(S\right)=\frac{1}{\alpha_{j}}d\left(R^{j},f\left(\phi \left(S\right)\right)\cdot P^{j}\right)-1$$

defines a weighted distance from the reference *j*, equivalent to the quality constraints. It is signed, with values in $\left[-1,\frac{1}{\alpha_{j}}-1\right]$ such that S is compatible with *i* iff $d_{j}\left(S\right)<0$ for j=i, and $d_{j}\left(S\right)\geq 0$ for all j≠i. Then, the QC process measures both the compatibility and the quality of S. The *compatibility* measures the normalized number of constraints satisfied for reference *i*:

(6) $$C_{i}\left(S\right)=\frac{1}{n_{\mathrm{ref}}}\left[1_{R_{<0}}\left(d_{i}\left(S\right)\right)+\sum_{j\neq i}1_{R_{\geq 0}}\left(d_{j}\left(S\right)\right)\right],$$

where $1_{X}\left(x\right)=\left\{\begin{array}{c} 1\;\text{if}\;x\in X\\ 0\;\text{if}\;x\notin X \end{array}\right)$ is the indicator function. S is *compatible* with *i* if $C_{i}\left(S\right)=1$. The *quality measure* provides more details by quantifying both the similarity with the reference *i* and the dissimilarity with the other references:

(7) $$Q_{i}\left(S\right)=\beta Q_{i}^{r}\left(S\right)+\left(1-\beta \right)Q_{i}^{\bar{r}}\left(S\right),$$

where $Q_{i}^{r}\left(S\right)$ is the quality measure between S and the reference on which compatibility is tested, i.e., the reference *i*:

(8) $$Q_{i}^{r}\left(S\right)=\max\left\{0,-d_{i}\left(S\right)\right\},$$

and $Q_{i}^{\bar{r}}\left(S\right)$ is the quality measure between S and other references:

(9) $$Q_{i}^{\bar{r}}\left(S\right)=\frac{1}{n_{\mathrm{ref}}-1}\sum_{j\neq i}\frac{\alpha_{j}}{1-\alpha_{j}}\max\left\{0,d_{j}\left(S\right)\right\},$$

with $\beta \in \left[01\right]$ a balancing coefficient. $Q_{i}\left(\cdot \right)\in \left[01\right]$, with $Q_{i}\left(S\right)=0$ in case no constraint is satisfied. As the distance vector, the compatibility and the quality measure encode different aspects of the quality, the process returns the tuple $\left(d,C_{i}\left(S\right),Q_{i}\left(S\right)\right)$. It can be directly used to check the quality of the preparation pipeline *i* or a preparation pipeline assumed to be similar to *i*.

#### Maximum quality reference

The QC process can also be used to determine if there is a reference preparation pipeline, and a corresponding processing pipeline, adapted to a given preparation pipeline S. We propose to retain a reference compatible with S and with maximum quality:

(10) $$\underset{\;\;\;\;\;\;i\in I}{\text{arg max}\;}Q_{i}\left(S\right),\;\text{with}\;\;I=\left\{k\in \left\{1,\ldots ,n_{\mathrm{ref}}\right\}\;|\;C_{k}\left(S\right)=1\right\}.$$

When S is not compatible with any reference ($C_{k}\left(S\right)<1$ for all *k*), a new reference should be integrated in the QC module, and validated as described in the next sections.

### Determining reference reduction matrices and weights

This section describes how the nuclei feature space is adapted to each reference WSI-PP, and how the reference weights are defined from these features.

#### Reduction matrices

For each reference preparation pipeline *i*, its reduction matrix $P^{i}$ is defined by selecting relevant features by a one-versus-all strategy, with regards to the other references. For this, we consider the decision problem (binary classification) which determines, for each nucleus of all references, whether the nucleus comes from the reference preparation *i* or not. Let $X\in R^{n_{\mathrm{ref}}n_{\mathrm{nuc}}\times n_{\text{feat}}}$ be the stack of all the nuclei feature matrices $X^{i}=\phi \left(S^{i}\right)$ obtained for all the references, and $y^{i}\in {\left\{01\right\}}^{n_{\mathrm{ref}}n_{\mathrm{nuc}}}$ be the associated nucleus binary labels, i.e.,

$$\forall k,\;y_{k}^{i}≔\left\{\begin{array}{cc} 1 & \text{if nucleus}\;k\;\text{comes from reference}\;i\\ 0 & \text{else}. \end{array}\right)$$

The pair $\left(\tilde{X},y^{i}\right)$, with ${\tilde{X}}_{k,l}=\frac{X_{k,l}-\min_{l}X_{k,l}}{\max_{l}X_{k,l}-\min_{l}\;X_{k,l}}$, is analyzed by a selection method $\phi_{\mathrm{sel}}$ that returns a $n_{\text{feat}}\times n_{\mathrm{sel}}$ selection matrix $P^{i}$, with $n_{\mathrm{sel}}\leq n_{\text{feat}}$ features:

(11) $$P^{i}=\phi_{\mathrm{sel}}\left(\tilde{X},y^{i},n_{\mathrm{sel}}\right).$$

For our experiments, the search space for $n_{\mathrm{sel}}$ were restricted to $\left\{10,20,50,100,\mathrm{all}=n_{\text{feat}}\right\}$, and two standard selection methods were tested. The first one is based on the chi-squared statistics.^3^ ^37^ The chi-squared test is performed independently for each feature, and the $n_{\mathrm{sel}}$ features receiving the lowest *p*-values are retained. The second selection method is based on minimum redundancy maximum relevance.4, ^53^ This method ranks features based on their importance, which is calculated as the quotient between features relevance and redundancy. Relevance is based on *F*-statistics, whereas redundancy is computed with Pearson correlation. The *n*_sel_ features receiving the highest importance score are retained. Thus, we considered 10 configurations for $\phi_{\mathrm{sel}}$, which are the combination of the two selection methods, denoted $\phi_{\mathrm{sel}}^{\mathrm{chi}2}$ and $\phi_{\mathrm{sel}}^{\text{mrmr}}$, with the five possible $n_{\mathrm{sel}}$ values.

#### Reference weights

**Fig. 4:**
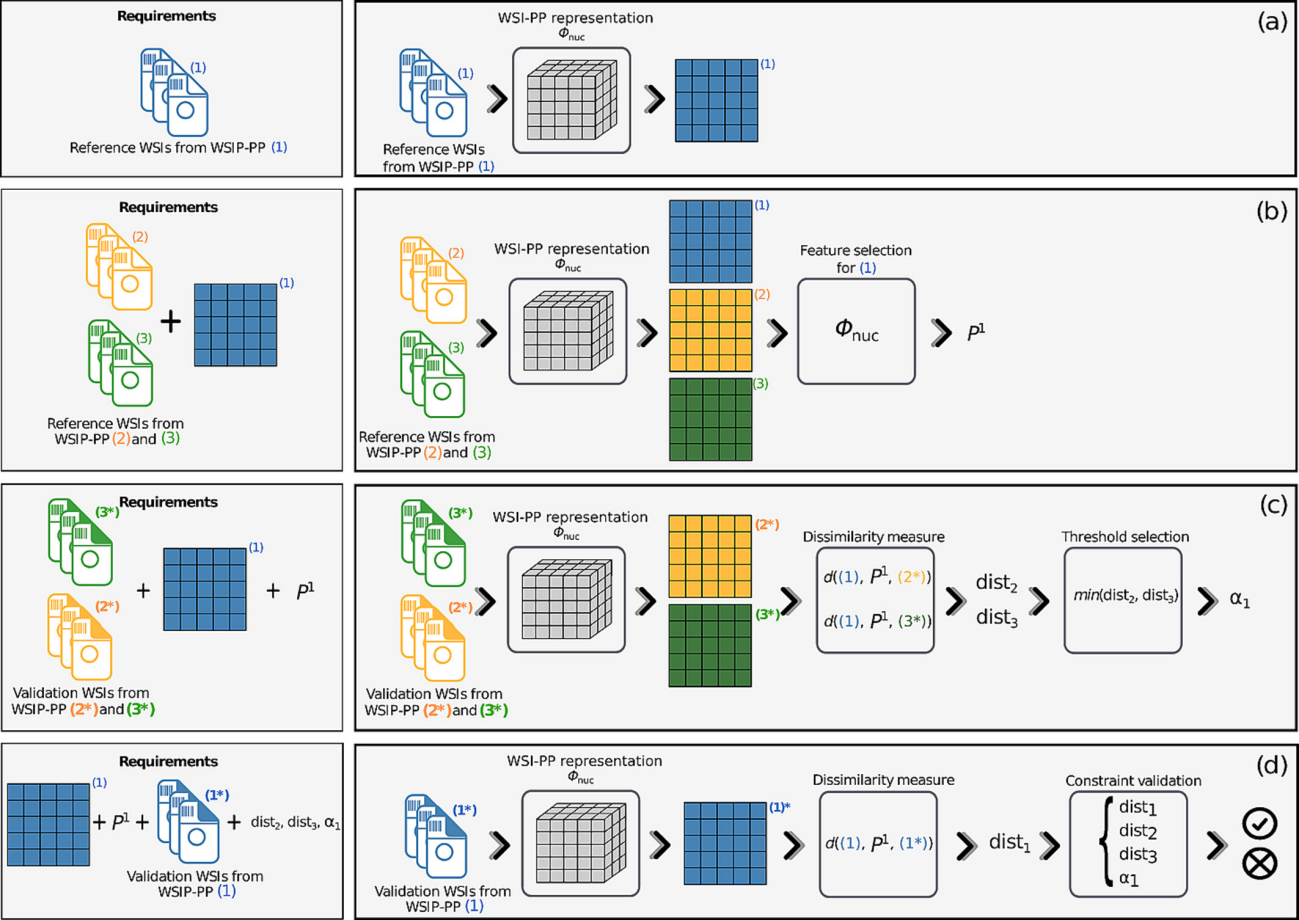
Stages to compute reference weights for a WSI-PP (1) with regards to WSI-PP (2) and (3). (a): Reference representation for WSI-PP (1) is computed. (b): Reduction matrix for WSI-PP (1) is obtained from reference representations of WSI-PPs (1), (2), and (3). (c): Distances between WSI-PPs validation (2*) and (3*) (which are pipelines with same characteristics than (2) and (3)) and WSI-PP reference (1) are computed. Reference weight for WSI-PP (1) is defined from these distances. (d): Distance between WSI-PP validation representation (1*) and WSI-PP reference representation (1) is computed. Constraints from Eqs. (3), (4) are checked in order to validate the reference weight.

The weights $\alpha_{i}$ involved in the quality constraints (Eqs. (3), (4)) and the quality process are defined by opposing the references to a validation set composed of $n_{\mathrm{val}}=n_{\mathrm{ref}}$ WSI-PPs. Each validation WSI-PP *i*, defined by a batch $V^{i}$ of $n_{\mathrm{wsi}}$ WSIs, is constrained to have characteristics similar to those of reference *i*. The reference minimum and maximum feature values already introduced, are defined as $m_{l}^{-}=\min_{i,k}\left\{V_{k,l}^{i},R_{k,l}^{i}\right\}$ and $m_{l}^{+}=\max_{i,k}\left\{V_{k,l}^{i},R_{k,l}^{i}\right\}$ for each nucleus feature *l*. By defining the weights by

(12) $$\forall i,\;\alpha_{i}≔\min_{j\neq i}\;\;d(R^{i},f\left(\phi \left(V^{j}\right)\right)\cdot P^{i}),$$

the quality constraint given by Eq. (4) is necessarily satisfied for all *i*. Contrary to that, the quality constraint given by Eq. (3) may not hold for all *i*. It is captured by the validation accuracy defined in the following. The entire stream to define reference weights is illustrated in Fig. 4.

### Validation process

This section presents the grid search approach we used to find relevant parameter configurations. It is based on a validation set $V={\left\{V^{i}\right\}}_{i}$ of WSI-PPs, as introduced in the previous section, and the accuracy and error metrics described below.

#### Parameters

The QC module parameters introduced so far are the number of WSIs $n_{\mathrm{wsi}}$, the nuclei selection method(s) $\phi_{\mathrm{nuc}}$ and the number of selected nuclei $n_{\mathrm{nuc}}$, the distance function ω and the aggregator *g*, the feature selection method $\phi_{\mathrm{sel}}$, and the number of reduced features $n_{\mathrm{sel}}$. As $\phi_{\mathrm{nuc}}$ methods are random (except for debris), the seed is also a parameter of the QC module. All parameters can take a finite number of values. Let $\Theta^{+}$ be the set of all possible parameter value combinations $\theta ={\left(\theta_{p}\right)}_{p=1,\ldots ,n_{\mathrm{par}}}$, with $n_{\mathrm{par}}=8$ the number of parameters. Note that the number of nuclei features $n_{\text{feat}}$ is not a parameter, it is fixed by the processing pipeline.

#### Accuracy

The accuracy of the QC module is defined as the sum of compatibilities (from Eq. 6):

(13) $$A≔\sum_{i=1}^{n_{\mathrm{ref}}}C_{i}\left(V^{i}\right)=\frac{1}{n_{\mathrm{ref}}}\sum_{i=1}^{n_{\mathrm{ref}}}1_{R_{<0}}\left(d_{i}\left(S\right)\right).$$

All validation WSI-PPs are compatible with their corresponding reference iff A=1 (i.e., Eq. 3 holds for all *i*). In this case, the QC module is considered as valid. We denote by $A_{\theta }$ the accuracy obtained for a specific parameter combination θ.

#### Error

Several combinations of parameter values can lead to a valid QC module (accuracy of 1). In order to differentiate them, we introduce a error function equivalent to the minimum quality reached by the WSI-PPs in *V* (Eq. 7). It is defined from normalized distances for more stability. Let $D\in {\left[01\right]}^{n_{\mathrm{ref}}\times n_{\mathrm{ref}}}$ be the distance matrix defined by $D_{i,j}≔d\left(R^{i},f\left(\phi \left(V^{j}\right)\cdot P^{i}\right)\right)$, and $\tilde{D}$ its normalized version defined by ${\tilde{D}}_{i,j}≔\frac{D_{i,j}-\mu_{j}^{-}}{\mu_{j}^{+}-\mu_{j}^{-}}$, with $\mu_{j}^{-}≔\min_{i}D_{i,j}$ and $\mu_{j}^{+}≔\max_{i}D_{i,j}$. Then, the error made by the QC module is measured by the error function:

(14) $$E≔1-\min_{i}\;\min_{j\neq i}\;{\tilde{D}}_{i,j}-{\tilde{D}}_{i,i}$$

The difference represents the margin before the quality constraint given by Eq. (3) is invalidated. We denote by $E_{\theta }$ the error obtained for a specific parameter combination θ.

#### Parameter selection

We describe the sketch of the grid search process used to find relevant parameters. Its inputs are the reference and validation WSI batches $R^{i}$ and $V^{i}$, respectively. As the number of combinations is large, in particular many seeds should be used to guarantee stable results, the search space is first reduced to a set Θ by setting both $n_{\mathrm{wsi}}$ and $n_{\mathrm{nuc}}$ to reasonable values, and by limiting to a unique seed. All the parameter values are listed in Table 1. Moreover, the impact of $\phi_{\mathrm{nuc}}^{\text{debris}}$ were analyzed in a separate experiment (reported in Appendix A.2). Then, a set of candidate combinations is determined in three main steps:

**Table 1:** List of tested parameters. Cumulative column (Cumul.) indicates whether the parameter can be used cumulatively or not.

Step	Parameter	Description	Values	Cumul.
Representation	$n_{\mathrm{wsi}}$	Number of WSIs	10, 20, 30	No
	$n_{\mathrm{nuc}}$	Number of nucleus	$10^{3},10^{4},10^{5}$	No
	$\phi_{\mathrm{nuc}}$	Nuclei selection method	$\phi_{\mathrm{nuc}}^{\text{intern},0.2}$, $\phi_{\mathrm{nuc}}^{\text{intern},0.4}$, $\phi_{\mathrm{nuc}}^{\text{distrib}}$	Yes
Measure	ω	Similarity function	$\omega_{\text{wass}}$, $\omega_{\text{bhat}}$, $\omega_{\text{jsha}}$	No
	g	Aggregator function	$g_{\min}$, $g_{\max}$, $g_{\mathrm{med}}$	No
Feature	$n_{\mathrm{sel}}$	Number of selected features	10, 20, 50, 100, all=$n_{\text{feat}}$	No
	$\phi_{\mathrm{sel}}$	Feature selection method	$\phi_{\mathrm{sel}}^{\text{mrmr}}$, $\phi_{\mathrm{sel}}^{\mathrm{chi}2}$	No

1. Select the valid combinations in Θ, i.e., the set $\Theta^{⋆}=\left\{\theta \in \Theta \;\right|A_{\theta }=1\}$.

2. Select at most $k_{1}\leq |\Theta^{⋆}|$ combinations in $\Theta^{⋆}$ that are the smallest according to the error:

$$\Omega \subseteq \Theta^{⋆}s.t.|\Omega |\leq k_{1}\;\text{and}\;\max_{\theta \in \Omega }E_{\theta }\leq \min_{\theta \in \Theta^{⋆}\backslash \Omega }E_{\theta }.$$

3. Select at most $k_{2}\leq |\Omega |$ combinations in Ω that are largest according to the objective defined by $T\left(\theta \right)=\left(1-E_{\theta }\right)+H\left(\theta \right)$ for any combination $\theta =\left(\theta_{1},\ldots ,\theta_{n_{\mathrm{par}}}\right)\in \Omega$, where

$$H\left(\theta \right)≔\sum_{p}h^{p}\left(\theta_{p}\right)$$

is the sum of parameter value frequencies in Ω, and $h^{p}\left(t\right)≔|\left\{\theta \in \Omega \;|\;\theta_{p}=t\right\}|/|\Omega |$ is the *t*-value frequency of the *p*-th parameter. The resulting set is given by

$$\Omega^{-}\subseteq \Omega \;s.t.|\Omega^{-}|\leq k_{2}\;\text{and}\;\min_{\theta \in \Omega^{-}}T\left(\theta \right)\geq \max_{\theta \in \Omega \backslash \Omega^{-}}T\left(\theta \right).$$

Then, the set of candidate combinations $\Omega^{-}$ is refined by examining the stability of the candidate combinations over several $n_{\text{seed}}$ new seeds. For each combination $\theta \in \Omega^{-}$, $n_{\text{seed}}$ combinations $\theta^{1},\ldots ,\theta^{n_{\text{seed}}}$ are created by replacing the initial seed value in θ by the new ones. The candidate combinations in $\Omega^{-}$ that remain valid for the $n_{\text{seed}}$ new seeds are retained as relevant:

$$\Omega^{⋆}=\left\{\theta \in \Omega^{-}|\sum_{s}A_{\theta^{s}}=n_{\text{seed}}\right\}.$$

These relevant combinations are used to determine a final combination, as well as the corresponding reference reduction matrices $P^{i}$ and weights $\alpha_{i}$. For this, accuracies and errors are analyzed for higher $n_{\mathrm{wsi}}$ and $n_{\mathrm{nuc}}$ values (detailed in the experiments section). The parameter search can only fail in case $\Theta^{⋆}$ is empty (step 1) or $\Omega^{⋆}$ is empty (refinement step). When this occurs, it is a QC alert that suggests to reconsider the reference and validation sets (e.g., WSI-PPs of poor quality) or the processing pipeline (e.g., unsuitable for some WSI-PPs). In the experiments we carried out, $\Theta^{⋆}$ and $\Omega^{⋆}$ were never empty, and the sizes of the sets Ω and $\Omega^{-}$ always corresponded to the expected $k_{1}$ and $k_{2}$ values.

### Materials

To compute reference weights for a set of WSI-PPs, we need access to a WSI-PP corpus as well as a nuclei analysis algorithm.

#### Analysis pipeline

Cytoprocessor,^9^ a cervical cancer screening system used by cytology laboratories to analyze nuclei, is used as the nuclei extraction algorithm. Analyzing a WSI via Cytoprocessor follows the steps depicted in Fig. 1. After detection and segmentation, 203 features are extracted per nucleus. These features are grouped into six categories: color,^38^ chromatin,^32^ gray level co-occurrence matrices,^15^ shape,^38^ and texture.^48^ These feature categories were chosen to optimize the performance of the subsequent analysis, which is carried out by a SVM-based classifier. It classifies nuclei into several classes, including those defined by the Bethesda system,^36^ in particular abnormal and debris classes. The debris class represents segmented objects not considered as nuclei by the classifier. It is used to define the nuclei selection method $\phi_{\mathrm{nuc}}^{\text{debris}}$ and to test the hypothesis that analyzing non-nuclei and mis-segmented nuclei are detrimental to QC.

#### WSI-PPs corpus

We consider seven WSI-PPs, denoted by pipeline_1_ to pipeline_7_. Each corpus is composed of 120 WSIs taken from a routine laboratory production that has undergone a Cytoprocessor validation, which consists of randomly taking 60 WSIs to confirm the Cytoprocessor diagnosis by a cytology expert. The main characteristics (preparation, coloration, and scanner type) of the WSI-PPs are summarized in Table 2. Each pipeline produces WSIs with distinct preparation characteristics that can be identified by an expert. Changes in preparation have the most significant impact on the characteristics of the WSIs, followed by changes in the scanner, and then changes in coloration. For instance, pipeline_4_ and pipeline_5_ are more difficult to distinguish because they only differ in their coloration stages, which are very similar. For each WSI-PP, we define the reference batch and the validation batch by randomly splitting the 120 WSIs into two batches of 60 WSIs each.

**Table 2:** WSIs preparation pipelines corpus description.

Pipeline	Preparation	Coloration	Scanner	Dim. (mm^2^)	Nb. nuclei (10^6^)
pipeline_1_	Surepath	Surepath	Pannoramic 1000	310	10.4
pipeline_2_	Thinprep	Thinprep	Pannoramic 1000	587	18.4
pipeline_3_	IDC20	ILSA	Pannoramic 1000	631	36.7
pipeline_4_	IDC20	ILSA	Pannoramic 250	549	25.3
pipeline_5_	IDC20	ILSA-diapath	Pannoramic 250	528	38
pipeline_6_	IDC20	ILSA	NanoZoomer S360	448	22.3
pipeline_7_	IDC20-C	ILSA	Aperio GT 450	579	37.9

#### Real-world corpus

To confront our QC module to real-world production environments, we dispose of cytology WSIs corpus from three laboratories, produced over periods of several weeks, as summarized in Table 3. For each corpus, no interval of more than four consecutive working days without production of WSIs is present.

**Table 3:** Routine laboratories WSI-PPs corpus description.

Laboratory	Pipeline identifier	Year	Production days	WSI number	Nuclei number in million
L	pipeline_2_	2020	33	3606	542
	pipeline_2_	2021	25	5162	752
C	pipeline_4_	2019	2	120	26
	pipeline_4_	2024	27	2220	723
M	pipeline_4_	2021	43	3656	749

## Experiments

In this section, we present the experiments carried out to find a relevant combination of parameter values for the proposed QC module. This set is found empirically by analyzing the results of the validation process. The following detail the application of this validation process to the corpus presented in Table 2.

A relevant combination of parameter values (Table 1) were computed by the validation process, with the validation and reference WSI sets, as defined in the validation process. The parameter values retained at each main stage are detailed in the following.

#### Relevant set of combinations $\Omega^{⋆}$

**Fig. 5:**
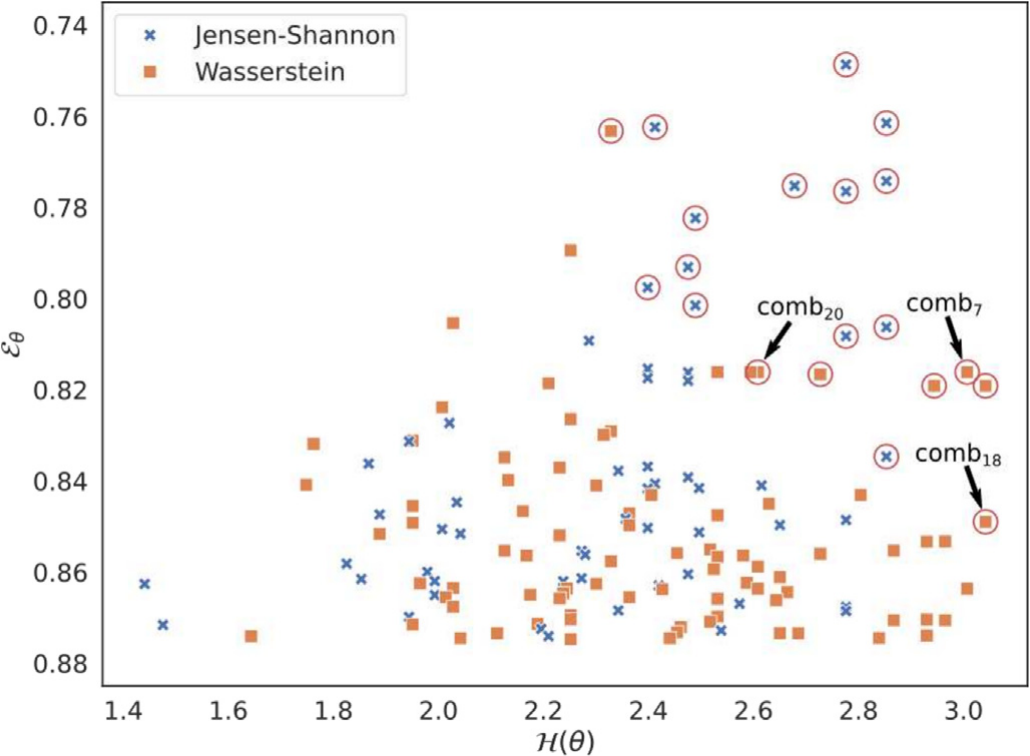
Relevant combinations according to H and 1−E. Circled combinations are those selected for $\Omega^{-}$. Combinations comb_20_, comb_7_ and comb_18_ are those retained for $\Omega^{⋆}$.

**Fig. 6:**
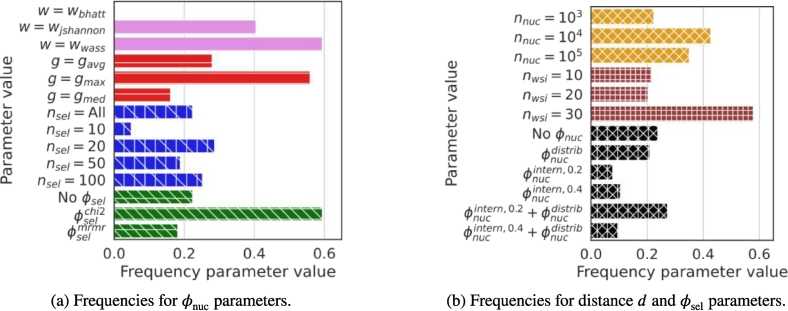
Frequency of the parameter values. Each style (color and marker) corresponds to a parameter.

In the first stage (steps 1–4), the number of WSIs is limited to $n_{\mathrm{wsi}}\in \left\{10,20,30\right\}$ and the number of nuclei to $n_{\mathrm{nuc}}\in \left\{10^{3},10^{4},10^{5}\right\}$. For steps 1–3, only one seed is used (for $\phi_{\mathrm{nuc}}$). This reduced the number of possible combinations to ∣Θ∣=4374. Then, the first step computes $|\Theta^{⋆}|=2860$ valid combinations, i.e., they comply with all quality constraints (top accuracy A=1). The second step refines the set of valid combinations by fixing $k_{1}=150$. Taking the 5 % combinations with lowest error values $E_{\theta }$ creates a set Ω of 143 combinations which all obtained an error below 0.875. The third step further reduces the number of combinations by selecting those that are best represented in Ω and minimize E, i.e., maximize T. For each parameter value *t*, its frequency $h^{p}\left(t\right)$ in Ω is shown in Fig. 6. We can observe that some parameter values are more frequent, i.e., the maximum aggregator $g_{\max}$, the Wasserstein distance $\omega_{\text{wass}}$, the nucleus selection method $\phi_{\mathrm{nuc}}^{\text{intern},0.2}+\phi_{\mathrm{nuc}}^{\text{distrib}}$ and the feature selection method $\phi_{\mathrm{sel}}^{\mathrm{chi}2}$. Moreover, a larger number of WSIs seems valuable. Also, we can observe that the frequencies for the number $n_{\mathrm{sel}}$ of reduced features is relatively homogeneous above 10. This suggests that $n_{\mathrm{sel}}=20$ could be sufficient. $\Omega^{-}$ is obtained by retaining $k_{2}=20$ combinations in Ω with highest $T_{\theta }$ values, as shown in Fig. 5. Finally, the refinement step generated a set $\Omega^{⋆}$ of 3 relevant combinations from $\Omega^{-}$ and 100 seeds (2000 combinations), refereed in Table 4. We observe that the best average results for the error $E_{\theta }$ are obtained with comb_20_. Moreover, none of the combinations with $\omega =\omega_{\mathrm{js}}$ was compatible on 100 seeds, which shows that the stability of our quality module with this distance is not sufficient. This can also be seen through the frequency parameter values (Fig. 5, Fig. 6), which are higher for $\omega_{\text{wass}}$, suggesting more stable compatibility results. We retained these 3 relevant combinations for the next step, which investigates the impact of several $n_{\mathrm{wsi}}$ and $n_{\mathrm{nuc}}$ on these combinations.

**Table 4:** Set of relevant combinations of parameter values from $\Omega^{⋆}$ that are compatible (i.e. obtained an $A_{\theta }$ of 1) for all seeds. For last two columns, ‘Avg.’ means average and ‘Std.’ means standard deviation.

Identifier	$n_{\mathrm{wsi}}$	$n_{\mathrm{nuc}}$	$\phi_{\mathrm{nuc}}$	ω	g	$\phi_{\mathrm{sel}}$	$n_{\mathrm{sel}}$	$T_{\theta }$	Avg. $\varepsilon_{\theta }$	Std. $\varepsilon_{\theta }$
comb_20_	30	10^4^	$\phi_{\mathrm{nuc}}^{\text{distrib}}+\phi_{\mathrm{nuc}}^{\text{intern},0.2}$	$\omega_{\text{wass}}$	$g_{\max}$	None	all	1.194	0.785	0.055
comb_7_	30	10^4^	$\phi_{\mathrm{nuc}}^{\text{distrib}}+\phi_{\mathrm{nuc}}^{\text{intern},0.2}$	$\omega_{\text{wass}}$	$g_{\max}$	$\phi_{\mathrm{sel}}^{\mathrm{chi}2}$	100	1.443	0.796	0.053
comb_18_	30	10^4^	$\phi_{\mathrm{nuc}}^{\text{intern},0.4}$	$\omega_{\text{wass}}$	$g_{\max}$	$\phi_{\mathrm{sel}}^{\mathrm{chi}2}$	20	1.204	0.835	0.054

#### Quantity of nuclei and WSIs

**Fig. 7:**
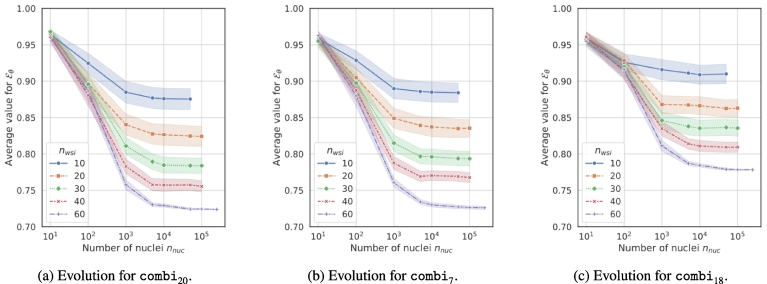
Evolution of error E for 100 seeds w.r.t. $n_{\mathrm{nuc}}$ and $n_{\mathrm{wsi}}$.

In the previous stages, the number of WSIs and nuclei were respectively limited to a maximum of $n_{\mathrm{wsi}}=30$ and $n_{\mathrm{nuc}}=10^{5}$. As larger values could reach better and more stable results, the last stage analyzes their impact while fixing the other parameter values as defined in Table 4. For this, $n_{\mathrm{wsi}}$ was incremented from 10 to 60 with a step of 10, and $n_{\mathrm{nuc}}\in \left\{10,10^{2},10^{3},5\cdot 10^{3},10^{4},5\cdot 10^{4},10^{5},5\cdot 10^{5}\right\}$ with a non-constant step. As before, the combinations were produced from 100 seeds. For 30 WSIs and 10^3^ nuclei, the accuracy were sufficiently high, with a null standard deviation. For the error, best results were obtained with 60 WSIs, with no gain in stability beyond 10^4^ nucleus, as observed in Fig. 7. So we have kept the latter values. To determine if the results from these three combinations, with $n_{\mathrm{wsi}}=60$ and $n_{\mathrm{nuc}}=10^{4}$, are significantly differentiable, we performed a Welch's *t*-test^5^ ^51^ with the null hypothesis that populations have same means, and a standard *p*-value of 0.05. We choosed a Welch's *t*-test as the resulting $E_{\theta }$ distributions followed a normal distribution without respecting homoscedasticity. Normality were checked with the Shapiro-Wilk test,^6^ ^42^ and homoscedasticity with the Bartlett's,^7^ ^45^ both with a *p*-value of 0.05. Results of comb_18_ are significantly differentiable of the other two combinations. However, results for comb_20_ and comb_7_ are not differentiable as they obtained a *p*-value of 0.057. The comb_20_ and comb_7_ only differ in the selection of features. The fact that feature selection has no impact on the results shows that some features are not relevant to our task, and can be removed without loss of performance. However, whatever the combination used between comb_20_ and comb_7_, the relevant set of features is present in it. Therefore, the most relevant feature is always selected via the max aggregator *g*_max_ We assume that with other types of aggregator, the selection of features could have a positive impact, as non-relevant features could be used in the aggregation process. We finally retained comb_20_ as the best combination because it does not use feature selection, thus saving computation time. We denote it comb^★^.

#### Reference WSI-PP reduction matrices and weights

As the combination comb^★^ does not use feature selection, all features were used, which amounts to assigning the identity to the reduction matrices, i.e., $P^{i}=I$ for any reference WSI-PP *i*. Weights $\alpha_{i}$ associated with comb^★^ are listed in Table 6. We observe two main groups with a relatively uniform weight distribution. For pipeline_4_ and pipeline_5_, the weights are very small compared to the other pipelines. This indicates that at least one other pipeline instantiated by the corresponding validation batch is very close to the reference according to the distance between distribution matrices. This is consistent with the preparation characteristics of the pipelines (Table 2). Indeed, in terms of these characteristics, pipeline_4_ and pipeline_5_ only differ in coloration, so they should be more difficult to distinguish. They are also close to pipeline_3_ and pipeline_6_, but with a different scanner. The pipelines 1, 2, and 3 share only the same scanner, and the pipelines 1 and 7 differ in at least two characteristics.

Validation was completed without any quality alerts. In order to identify potential quality issues, the retained combination (Table 5), and associated elements representing references (feature distributions, reduction matrices, weights) were used to test the QC module on large routine corpus from different laboratories.

**Table 5:** Combination comb^⋆^ of relevant parameter values retained by the validation process.

$n_{\mathrm{wsi}}$	$n_{\mathrm{nuc}}$	$\phi_{\mathrm{nuc}}$	ω	g	$\phi_{\mathrm{sel}}$	$n_{\mathrm{sel}}$
60	10^4^	$\phi_{\mathrm{nuc}}^{\text{distrib}}$ + $\phi_{\mathrm{nuc}}^{\text{intern},0.2}$	$\omega_{\text{wass}}$	$g_{\max}$	None	all

**Table 6:** Reference WSI-PP weights $\alpha_{i}$ associated with combination comb^★^ (Table 5).

pipeline_1_	pipeline_2_	pipeline_3_	pipeline_4_	pipeline_5_	pipeline_6_	pipeline_7_
0.1868	0.1266	0.1292	0.0982	0.0809	0.1345	0.1720

## Discussion

In this section, we discuss results of the QC module, created from combination comb^★^ (see Table 5) and reference WSI-PP corpus described in the materials section, on real-world production environments from Table 3. To ensure QC for each laboratory WSI-PP stream, the quality module is executed on batches of 60 WSIs. Batches are contiguous in time and don't share common WSIs.

For each batch, the compatibility measure (Eq. 6) and the quality measure (Eq. 7) with β=0 are performed. Despite the fact that the results of these two measures show the same trend, the compatibility measure seems to be too strict, as very few batches are compatible with a pipeline. On the other hand, the quality measure seems to correctly identify the valid pipeline, expect for laboratory C. More details on these results can be found in Table A.2, Table A.3, Table A.4 of the Appendix. In order to strike a balance between these two measures, we have chosen to present and interpret in this section the results of the quality measure with β=0.7.

### Laboratory L

**Fig. 8:**
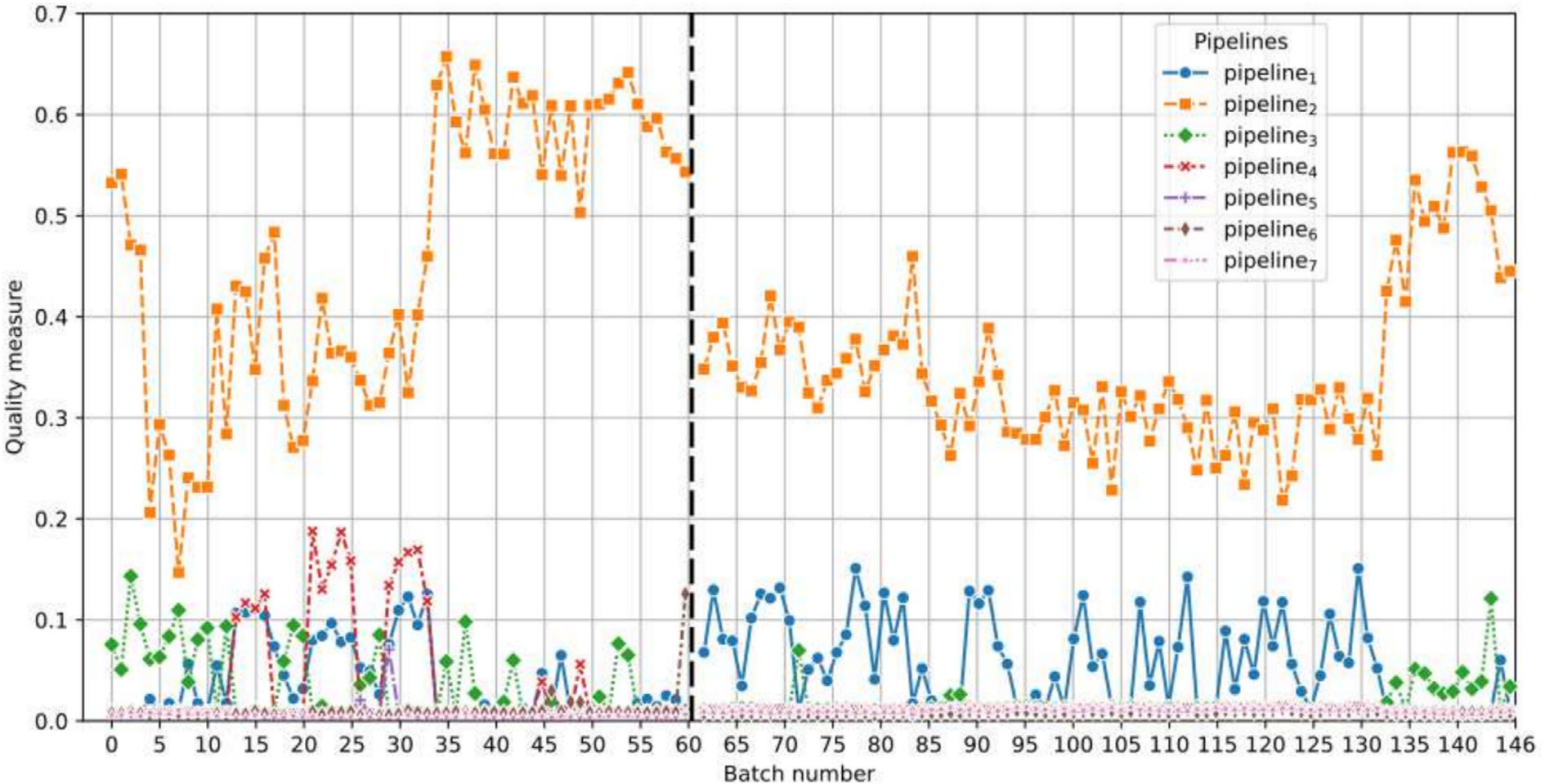
Quality measure for each representative pipeline with respect to the batches created for laboratory L. The horizontal dotted black bar is used to separate 2020 and 2021 corpus.

Two streams of WSIs from different timelines in laboratory L are analyzed. The first stream, comprising 3606 WSIs, was produced in 2020, whereas the second stream, consisting of 5162 WSIs, was produced in 2021. Because the WSI-PP of laboratory L shares the same characteristics as pipeline_1_ (including preparation, coloration, and scanner), it is expected that the reference for pipeline_1_ yields a higher quality measure compared to the others. As anticipated, the quality measure with respect to pipeline_1_ is the highest. Additionally, the measure remains stable and consistent across all batches and both years, indicating quality stability in the WSI-PP, as illustrated in Fig. 8.

### Laboratory C

The data used to depict laboratory C consists of two streams of WSIs from two different timelines. The first stream comprises 120 WSIs produced in 2019, whereas the second stream consists of 2220 WSIs produced in 2024. As laboratory C's WSI-PP shares the same characteristics as pipeline_4_, it is expected that the algorithm for pipeline_4_ will yield a higher quality measure compared to the others. Moreover, because pipeline_4_ and pipeline_5_ are almost identical (see Table 2), the quality measures with respect to pipeline_5_ are also anticipated to be higher than the others.

**Fig. 9:**
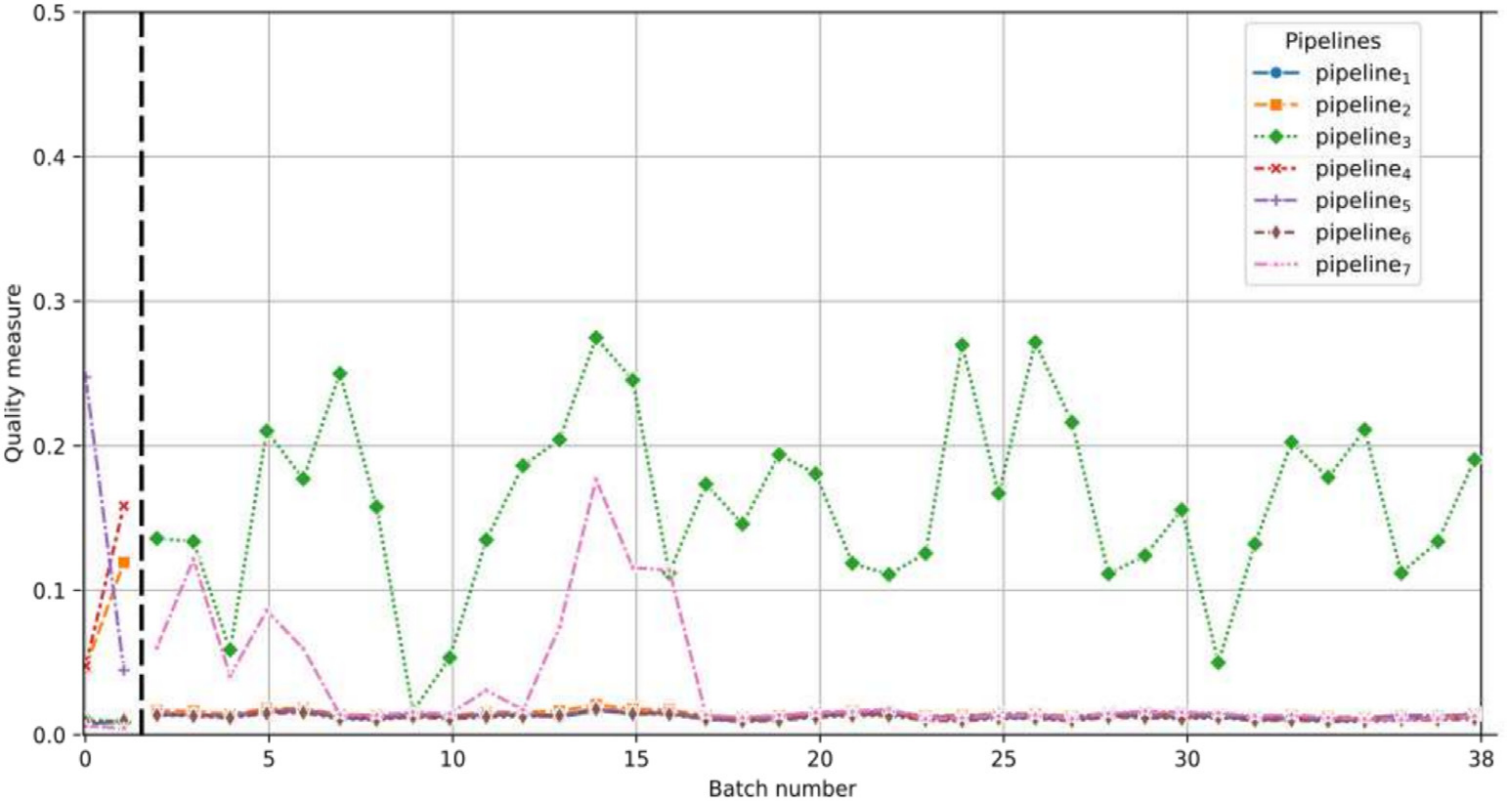
Quality measure for each representative pipeline with respect to the batches created for laboratory C. The horizontal dotted black bar is used to separate 2019 and 2024 corpus.

As depicted in Fig. 9, expected quality measures for the 2019 batches are observed, as score for pipeline_4_ and pipeline_5_ are among the three best measures. However, for 2024 batches, the quality measure for the pipeline_4_ and pipeline_5_ is no longer among the best measures, alerting us to a potential quality problem.

As our dissimilarity measure employs the max aggregator function *g*_max_, the final dissimilarity distance is determined by a single feature, allowing for easier interpretation of results. In our case, to differentiate the 2019 and 2024 streams from pipeline_4_, we have established that features characterizing green colors are used for all batches. One example of such a feature is *green_min*, which represents the minimum value of green within a nucleus.

**Fig. 10:**
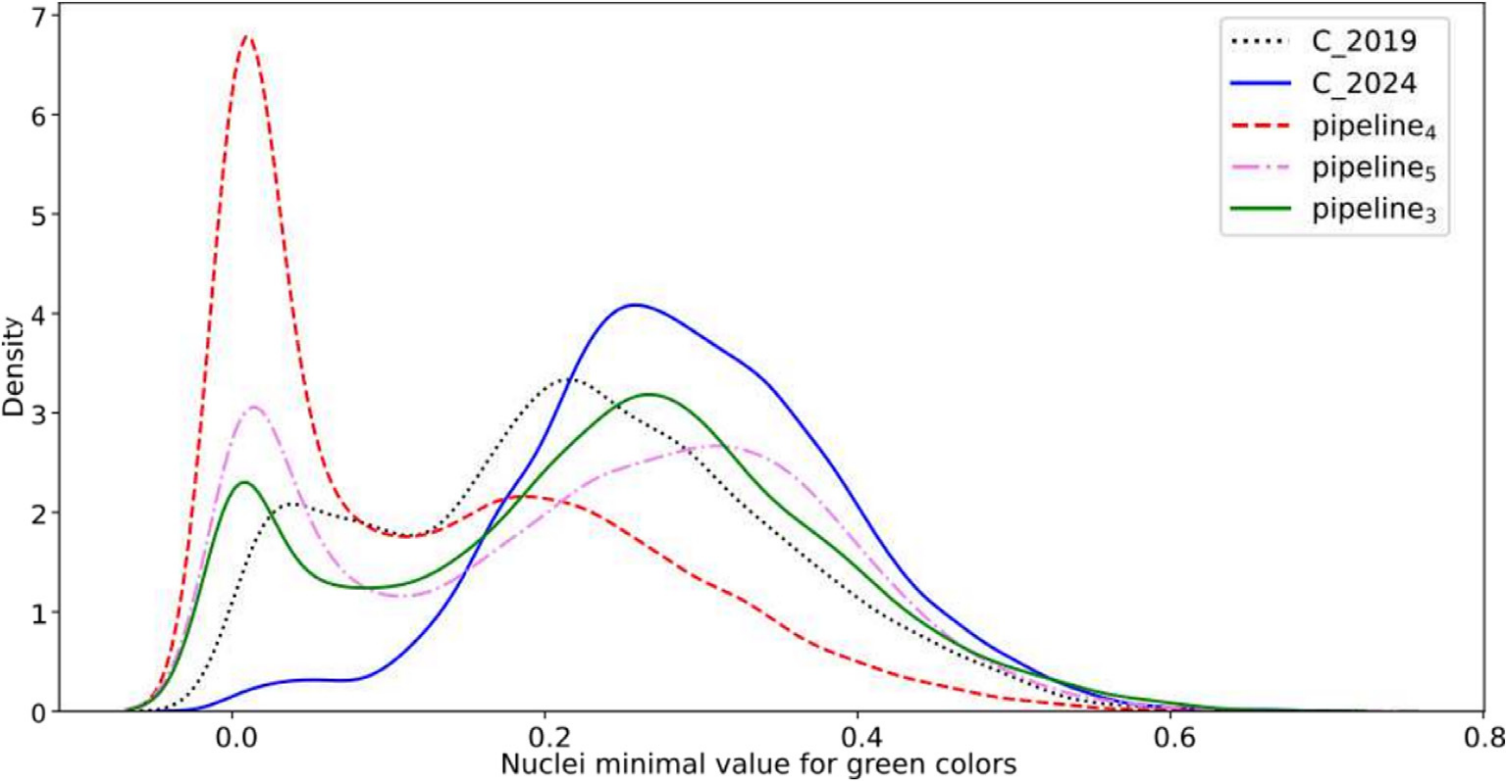
Continuous probability density curve for feature *green_*min. Curves obtained by fitting a kernel density estimate on the observed feature values.

This difference in green can be observed by comparing the distribution of values across random batches from 2019, 2024, pipeline_3_, pipeline_4_, and pipeline_5_. Fig. 10 illustrates this by plotting the continuous probability density curves of the pipelines, obtained using a kernel density estimate.^8^

**Fig. 11:**
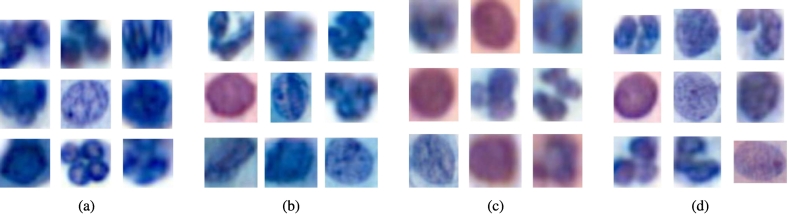
Nuclei comparison between laboratory C, pipeline_4_ and pipeline_3_. Nuclei with the median value for feature *green_min* have been chosen for pipeline_4_ (a), laboratory C pipeline 2019 (b), laboratory C pipeline 2024 (c) and pipeline_3_ (d).

Additionally, this difference is visually evident, as the nuclei in pipeline_4_ appear darker than those from 2019, which in turn are darker than the nuclei from the 2024 pipeline, as shown in Fig. 11.

### Laboratory M

For laboratory M, we have 3656 WSIs produced in the final months of 2021. Similar to the previous laboratory, laboratory M's WSI-PP shares the same characteristics as pipeline_4_. Therefore, quality measures with respect to pipeline_4_ and pipeline_5_ are expected to be maximized, whereas others should be inferior.

**Fig. A.3:**
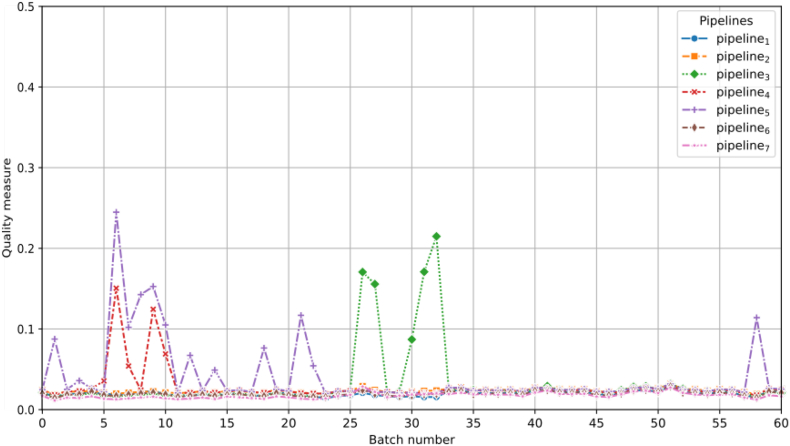
Quality measure for each pipeline of our metric with respect to the batches created for laboratory M.

In initial experiments, we observed that none of the tested pipelines achieved satisfactory quality measurements (see Fig. A.3 in the Appendix for more details). Given the similarity between pipeline_4_ and pipeline_5_, we hypothesized that these pipelines come into conflict during their reference weight definition. Consequently, we redefined the weights by merging these two pipelines (see Table A.5 in the Appendix). Note that the parameter values used to define these new weights, specifically those from combination comb^★^, are not necessarily optimal because they were determined considering pipeline_4_ and pipeline_5_ as distinct entities.

**Fig. 12:**
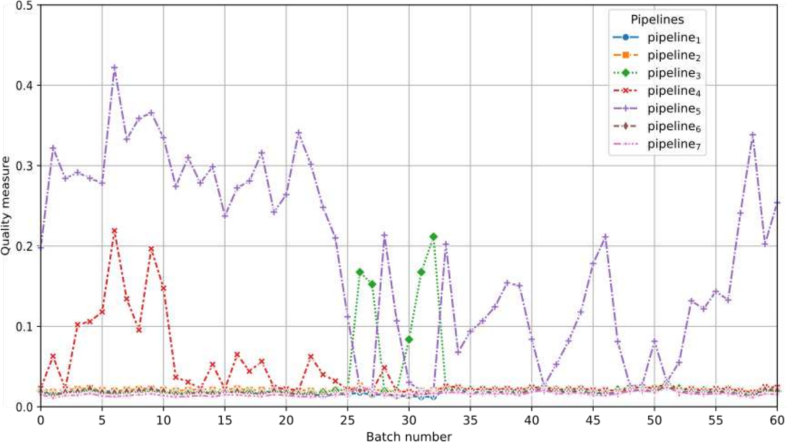
Quality measure for each representative pipeline with respect to the batches created for laboratory M, when considering pipeline 4 and 5 as same.

**Fig. 13:**
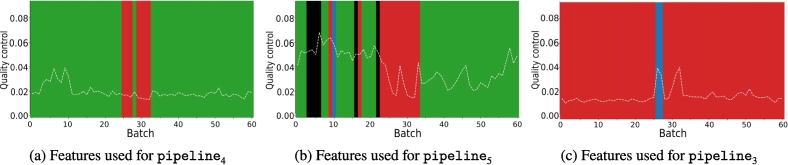
Features used for the quality measure of laboratory M w.r.t. to different WSI-PP. Features characterizing colors are represented by the respective color (green, red, or blue), whereas others are represented in black. Quality measure is represented via the dotted line. (For interpretation of the references to color in this figure legend, the reader is referred to the web version of this article.)

**Fig. 14:**
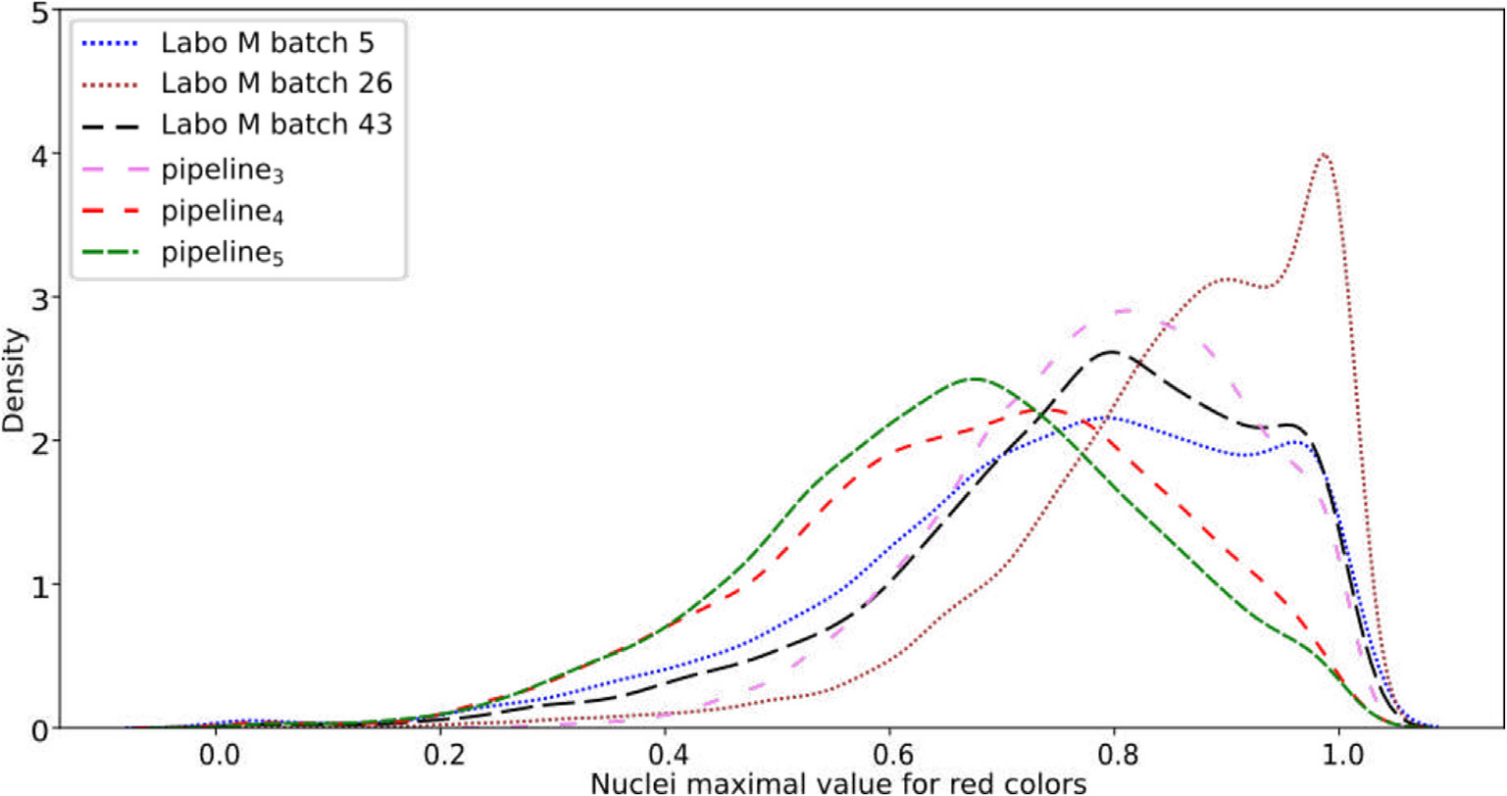
Continuous probability density curve for feature *red_max*. Curves obtained by fitting a kernel density estimate on the observed feature values.

**Fig. 15:**
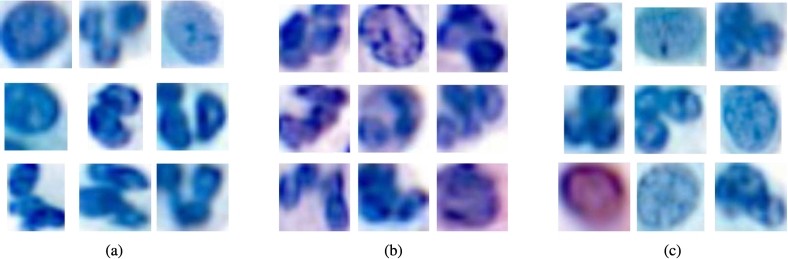
Set of nuclei images for laboratory M. Nuclei with the median value for feature *red_max* have been chosen for 3 batches of laboratory M pipeline: (a) batch 5; (b) batch 26; (c) batch 43.

With these new weights, we observed that pipeline_4_ still did not yield the expected results, whereas pipeline_5_ was validated for all batches except those between batch 26 and 33 (see Fig. 12). Fig. 13 shows the features used by the quality measure to differentiate pipeline M from pipeline_2_, pipeline_4_, and pipeline_5_. The features used remain generally consistent, except between batch 26 and 33, where a change in the nuclei red colors appears to be present. This change can be observed by examining the distributions for the feature *red_max* across different pipelines (see Fig. 14), where a noticeable alteration in the shape of the batch 26 curve is evident compared to previous and subsequent batches. Furthermore, this color deviation is visible upon direct inspection of the nuclei, as depicted in Fig. 15, where the nuclei from batch 26 appear redder (or more violet) than those from the preceding and following batches during the validation process.

**Fig. 16:**
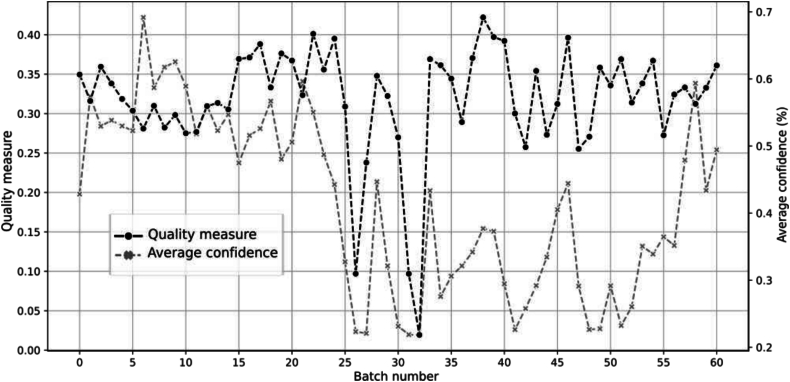
Relation between our quality measure and algorithm's confidence.

To evaluate the relevance of our quality measure with respect to the algorithm's performance, we compared our measure to the confidence levels of the classification algorithm's predictions, specifically using an SVM. We observed a correlation between the average batch classification confidences and the batch quality measures, as shown in Fig. 16. This observation suggests that the deviations detected by our measure are likely detrimental to the algorithm's performance.

## Conclusion

In this article, we outlined the need for QC from the algorithmic point of view in DP, and particularly in cytology, where preparation errors can be caused by many variables. To address this, we introduced a QC module to ensure compatibility of a preparation pipeline with diverse algorithms. In contrast to prevalent methods, our approach performs QC from the algorithmic perspective, requires minimal computational resources and offers ease to interpretation.

Through empirical analysis, conducted on seven distinct WSI-PPs, we demonstrated that our method clearly discriminates each pipeline. Additionally, we validated its performance on larger and realistic corpus, detecting quality deviations and highlighting a correlation between the quality measure and the algorithm's confidence.

Whereas our method excels in its simplicity and efficiency, there are avenues for enhancement. Introducing a trainable aggregator function for the dissimilarity measure seems promising, despite it may reduce interpretability. Furthermore, adopting a dissimilarity function capable of measuring distributions with multiple dimensions could better capture the nuances, probably at the cost of greater computing power. We strongly believe in the necessity of QC from the algorithmic perspective and hope this kind of approach will be extended to other WSI representations, technologies and datasets, thereby advancing the quality and reliability of DP WSIs.

## Funding sources

The work of Paul Barthe is partly supported by the 10.13039/501100003032ANRT with Cifre number 2023/0122.

## Declaration of competing interest

The authors declare the following financial interests/personal relationships which may be considered as potential competing interests: Paul Barthe, Romain Brixtel, Yann Caillot, Benoît Lemoine, Arnaud Renouf, Vianney Thurotte and Ouarda Beniken were employed at Datexim during this study.

## Figures and Tables

**Fig. A.1:**
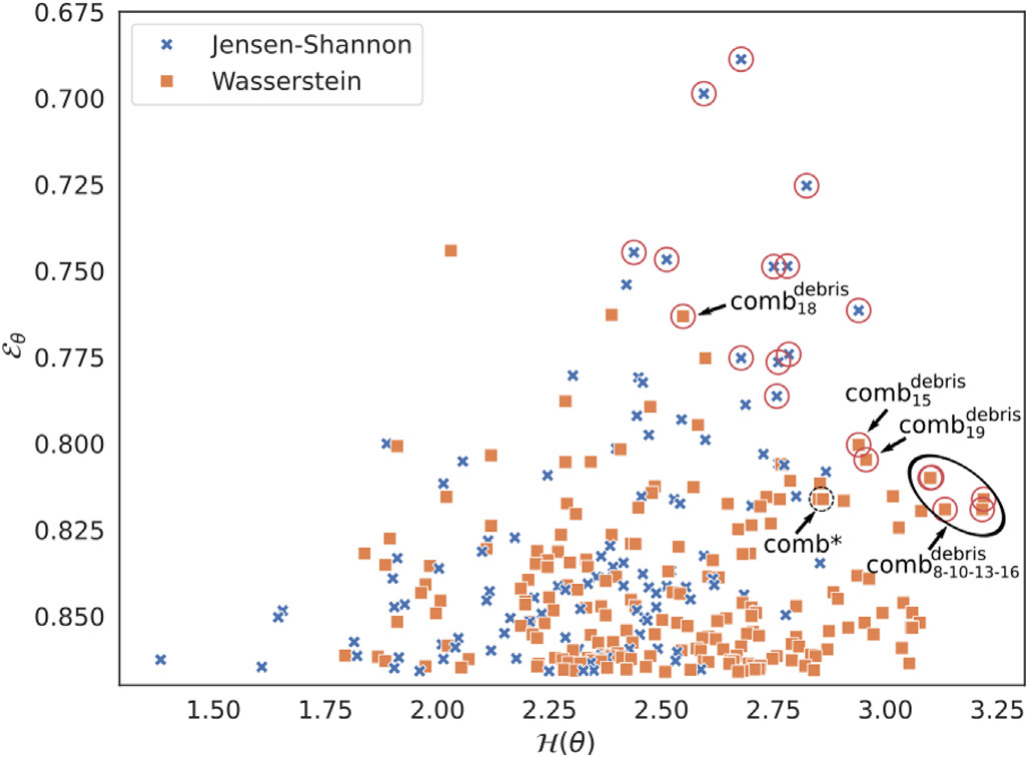
Relevance of combinations, including $\phi_{\mathrm{nuc}}^{\text{debris}}$ parameter, according to H and E. Circled combinations are those selected for $\Omega^{-}$. Combination comb^★^ is selected after Section 4 validation process.

**Table A.1:** Set of relevant combinations of parameter values from $\Omega^{⋆}$ that are compatible (i.e. obtained an $A_{\theta }$ of 1) for all seeds. Set obtained with $\phi_{\mathrm{nuc}}^{\text{debris}}$ activated. For last two columns, ‘Avg.’ means average and ‘Std.’ means standard deviation.

Identifier	$n_{\mathrm{wsi}}$	$n_{\mathrm{nuc}}$	$\phi_{\mathrm{nuc}}$	ω	g	$\phi_{\mathrm{sel}}$	$n_{\mathrm{sel}}$	$T_{\theta }$	Avg. $E_{\theta }$	Std. $E_{\theta }$
$\mathrm{com}b_{18}^{\text{debris}}$	30	10^4^	$\phi_{\mathrm{nuc}}^{\text{distrib}}+\phi_{\mathrm{nuc}}^{\text{intern},0.2}$	$\omega_{\text{wass}}$	$g_{\mathrm{avg}}$	None	all	1.215	0.77	0.058
$\mathrm{com}b_{8}^{\text{debris}}$	30	10^4^	$\phi_{\mathrm{nuc}}^{\text{distrib}}+\phi_{\mathrm{nuc}}^{\text{intern},0.2}$	$\omega_{\text{wass}}$	$g_{\max}$	$\phi_{\mathrm{sel}}^{\mathrm{chi}2}$	100	1.282	0.796	0.053
$\mathrm{com}b_{13}^{\text{debris}}$	30	10^4^	$\phi_{\mathrm{nuc}}^{\text{distrib}}+\phi_{\mathrm{nuc}}^{\text{intern},0.2}+\phi_{\mathrm{nuc}}^{\text{debris}}$	$\omega_{\text{wass}}$	$g_{\max}$	$\phi_{\mathrm{sel}}^{\mathrm{chi}2}$	100	1.254	0.815	0.034
$\mathrm{com}b_{19}^{\text{debris}}$	30	10^5^	$\phi_{\mathrm{nuc}}^{\text{intern},0.2}$+$\phi_{\mathrm{nuc}}^{\text{debris}}$	$\omega_{\text{wass}}$	$g_{\max}$	$\phi_{\mathrm{sel}}^{\mathrm{chi}2}$	100	1.203	0.817	0.036
$\mathrm{com}b_{16}^{\text{debris}}$	30	10^4^	$\phi_{\mathrm{nuc}}^{\text{distrib}}+\phi_{\mathrm{nuc}}^{\text{intern},0.2}$	$\omega_{\text{wass}}$	$g_{\max}$	$\phi_{\mathrm{sel}}^{\mathrm{chi}2}$	50	1.218	0.819	0.061
$\mathrm{com}b_{10}^{\text{debris}}$	30	10^4^	$\phi_{\mathrm{nuc}}^{\text{distrib}}+\phi_{\mathrm{nuc}}^{\text{intern},0.2}$	$\omega_{\text{wass}}$	$g_{\max}$	$\phi_{\mathrm{sel}}^{\mathrm{chi}2}$	20	1.263	0.819	0.064
$\mathrm{com}b_{15}^{\text{debris}}$	30	10^3^	$\phi_{\mathrm{nuc}}^{\text{distrib}}+\phi_{\mathrm{nuc}}^{\text{intern},0.2}$+$\phi_{\mathrm{nuc}}^{\text{debris}}$	$\omega_{\text{wass}}$	$g_{\max}$	$\phi_{\mathrm{sel}}^{\mathrm{chi}2}$	100	1.219	0.847	0.039

**Fig. A.2:**
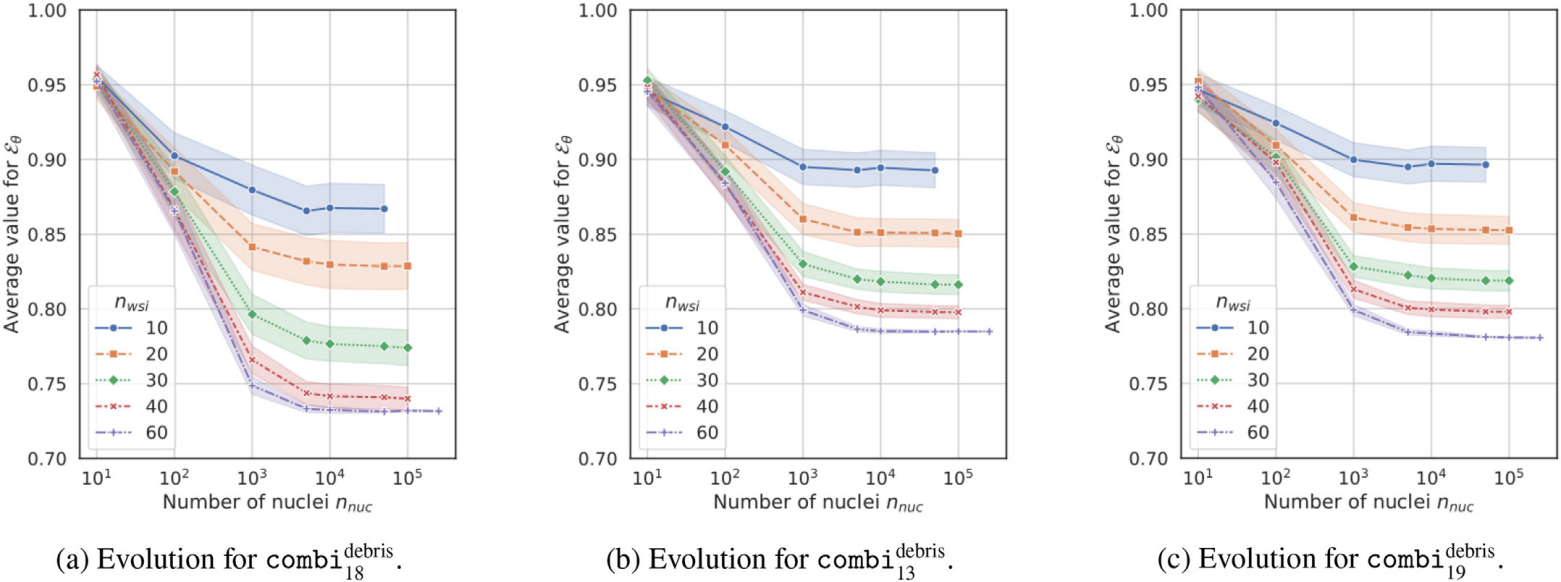
Evolution of the error E for 100 seeds and for debris combinations, w.r.t. $n_{\mathrm{nuc}}$ and $n_{\mathrm{wsi}}$.

**Table A.2:** Percentage of laboratory L batches that verified Eq. (6) (second column) or that obtained each possible accuracy score (third column), w.r.t. to the all pipelines. Lines in bold correspond to pipelines designed for the laboratory.

Pipeline name	Eq. 3 verified	Accuracy scores							
		0/7	1/7	2/7	3/7	4/7	5/7	6/7	7/7
pipeline_1_	67.35 %	0	0 %	0 %	1.36 %	23.13 %	27.89 %	47.62 %	0 %
pipeline_2_	**100 %**	**0 %**	**0 %**	**0 %**	**0.68 %**	**2.04 %**	**17.69 %**	**68.71 %**	**10.88 %**
pipeline_3_	29.93 %	0 %	0 %	1.36 %	9.52 %	49.66 %	19.73 %	19.73 %	0 %
pipeline_4_	11.56 %	0 %	0 %	0 %	9.52 %	70.75 %	19.73 %	0 %	0 %
pipeline_5_	1.36 %	0 %	0 %	1.36 %	18.37 %	69.39 %	10.88 %	0 %	0 %
pipeline_6_	2.72 %	0 %	0.68 %	1.36 %	17.01 %	68.03 %	11.56 %	1.36 %	0 %
pipeline_7_	0 %	0 %	0.68 %	2.04 %	17.69 %	68.71 %	10.88 %	0 %	0 %

**Table A.3:** Percentage of laboratory C batches that verified eq. 6 (second column) or that obtained each possible accuracy score (third column), w.r.t. to the all pipelines. Lines in bold correspond to pipelines designed for the laboratory.

Pipeline name	Eq. 3 verified	Accuracy scores							
		0/7	1/7	2/7	3/7	4/7	5/7	6/7	7/7
pipeline_1_	0 %	0.0 %	0.0 %	0.0 %	0.0 %	27.03 %	70.27 %	2.7 %	0.0 %
pipeline_2_	5.13 %	0.0 %	0.0 %	0.0 %	0.0 %	27.03 %	70.27 %	2.7 %	0.0 %
pipeline_3_	92.31 %	0.0 %	0.0 %	0.0 %	0.0 %	0.0 %	0.0 %	29.73 %	70.27 %
pipeline_4_	**5.13 %**	**0.0 %**	**0.0 %**	**0.0 %**	**0.0 %**	**27.03 %**	**70.27 %**	**2.7 %**	**0.0 %**
pipeline_5_	**5.13 %**	**0.0 %**	**0.0 %**	**0.0 %**	**0.0 %**	**27.03 %**	**70.27 %**	**2.7 %**	**0.0 %**
pipeline_6_	0 %	0.0 %	0.0 %	0.0 %	0.0 %	27.03 %	70.27 %	2.7 %	0.0 %
pipeline_7_	25.64 %	0.0 %	0.0 %	0.0 %	0.0 %	0.0 %	70.27 %	29.73 %	0.0 %

**Table A.4:** Percentage of laboratory M batches that verified eq. 6 (second column) or that obtained each possible accuracy score (third column), w.r.t. to the all pipelines. Lines in bold correspond to pipelines designed for the laboratory.

Pipeline name	Eq. 3 verified	Accuracy scores							
		0/7	1/7	2/7	3/7	4/7	5/7	6/7	7/7
pipeline_1_	0 %	0.0 %	0.82 %	0.0 %	15.57 %	45.9 %	34.43 %	3.28 %	0.0 %
pipeline_2_	0 %	0.0 %	0.0 %	0.0 %	6.56 %	39.34 %	44.26 %	9.84 %	0.0 %
pipeline_3_	8.2 %	0.0 %	0.0 %	17.21 %	10.66 %	26.23 %	36.89 %	5.74 %	3.28 %
pipeline_4_	**31.15 %**	**0.0 %**	**0.0 %**	**5.74 %**	**18.03 %**	**22.95 %**	**32.79 %**	**20.49 %**	**0.0 %**
pipeline_5_	**86.89 %**	**0.0 %**	**0.0 %**	**0.82 %**	**9.02 %**	**27.05 %**	**14.75 %**	**21.31 %**	**27.05 %**
pipeline_6_	0 %	0.0 %	5.74 %	22.13 %	14.75 %	22.13 %	30.33 %	4.92 %	0.0 %
pipeline_7_	0 %	0.0 %	6.56 %	22.95 %	13.93 %	22.95 %	30.33 %	3.28 %	0.0 %

**Table A.5:** Reference WSI-PP weights $\alpha_{i}$ associated with combination comb^★^ (Table 5) without considering pipeline_4_ and pipeline_5_ as distinct WSI-PPs.

pipeline_1_	pipeline_2_	pipeline_3_	pipeline_4_	pipeline_5_	pipeline_6_	pipeline_7_
0.1859	0.1263	0.1297	0.1118	0.1271	0.1337	0.1730

## Appendix A Appendix

### A.1 Experiments

#### A.1.1 Implementation details

Detection, segmentation, and features calculation were run on an Intel Xeon Silver 4214R Processor (max turbo frequency of 3.5 GHz). Experiments were run on an Intel Core i9-12900K Processor (max turbo frequency of 5.20 GHz).

#### A.1.2 Calculation time

The average time required to detect, segment, and compute features to nuclei on one WSI is 12 min. The time required to calculate reference weights with optimal parameters found in our experiments, for a corpus of seven pipelines, is less than 5 min. The time required to test the quality of a pipeline w.r.t. to the seven pipelines contained in our measure, with optimal parameters found in our experiments, is also less than 5 min.

### A.2 Validation process with debris class

The validation process described applied in experiments is repeated with one more parameter value for $\phi_{\mathrm{nuc}}$: $\phi_{\mathrm{nuc}}^{\text{debris}}$. Has already explained, this selection method removes segmented objects not considered as nuclei by Cytoprocessor classifier. Note that $\phi_{\mathrm{nuc}}^{\text{debris}}$ is only intended to test the hypothesis that artifacts or failed segmentations are detrimental to QC. Indeed, as the QC module is placed before the analysis, the classification results are inaccessible for Cytoprocessor-based QC in practice.

#### A.2.1 Relevant set of combinations $\Omega^{⋆}$

We fixed $n_{\mathrm{wsi}}=30$, $n_{\mathrm{nuc}}=10^{5}$ and limit the grid search to one seed for step 1 to 3, which reduced the number of possible combinations to ∣Θ∣=8748. The first step of the validation process computed $|\Theta^{⋆}|=6015$ valid combinations. The second step reduced the set of valid combinations to a set Ω of 301 combinations, all with an error below 0.866. Then, $\Omega^{-}$ is obtained in the third step by retaining $k_{2}=20$, as shown in Fig. A.1. Finally, the refinement step generated a set $\Omega^{⋆}$ of 7 relevant combinations refereed in Table A.1. We observe that the 3 best average results for the error $E_{\theta }$ were obtained with $\mathrm{com}b_{18}^{\text{debris}}$, $\mathrm{com}b_{8}^{\text{debris}}$ and $\mathrm{com}b_{13}^{\text{debris}}$. As $\mathrm{com}b_{8}^{\text{debris}}$ is equivalent to $\mathrm{com}b_{7}$ of experiments section, it was not considered for the next step. Therefore, we retained $\mathrm{com}b_{18}^{\text{debris}}$, $\mathrm{com}b_{13}^{\text{debris}}$, and $\mathrm{com}b_{19}^{\text{debris}}$ to investigate the impact of higher $n_{\mathrm{wsi}}$ and $n_{\mathrm{nuc}}$.

#### A.2.2 Quantity of nuclei and WSIs

To analyze the impact of nuclei and WSIs quantity, $n_{\mathrm{wsi}}$ and $n_{\mathrm{nuc}}$ were incremented as in the experiments section. For 30 WSIs and 10^3^ nuclei, the accuracy were sufficiently high, with a null standard deviation. For the error, best results were obtained with 60 WSIs, with no gain in stability beyond 10^4^ nucleus, see in Fig. A.2. With this quantity of WSIs and nuclei, best results were obtained by ${\mathrm{comb}}_{18}^{\text{debris}}$ that reached an average E of 0.732. To determine if these results, with $n_{\mathrm{wsi}}=60$ and $n_{\mathrm{nuc}}=10^{4}$, are significantly differentiable, we performed a Welch's *t*-test as the resulting $E_{\theta }$ distributions followed a normal distribution without respecting homoscedasticity. This test was performed with a standard *p*-value of 0.05. The results of ${\mathrm{comb}}_{18}^{\text{debris}}$ are significantly different from the other two combinations. However, the results for ${\mathrm{comb}}_{18}^{\text{debris}}$ and $\mathrm{com}b^{⋆}$ are not differentiable because they obtained a *p*-value of 0.117. This suggests that debris objects are useful, or at least not disruptive, to discriminate our WSI-PPs (Table 2).

### A.3 Quality control in routine laboratories

#### A.3.1 Additional details

Table A.2, Table A.3, Table A.4 detail the number of batches that have validated the quality module constraints, for laboratory L, C, and M. More precisely, they detail the percentage of batches that validate Eq. (3) alone, and the percentage of batches that reached each possible accuracy score from Eq. (13). Fig. A.3 shows the results obtained for laboratory M batches with reference weights obtained by considering reference pipeline_4_ and pipeline_5_ as distinct. These weights are specified in Table A.5.

## Footnotes

1 SciPy implementation: scipy.stats.wasserstein_distance of SciPy v1.11.3.

2 SciPy implementation: scipy.spatial.distance.jensenshannon of SciPy v1.11.3.

## Footnotes

3 Scikit-learn implementation: sklearn.feature_selection.chi2 of Scikit-learn v1.2.2.

4 Implementation: https://github.com/smazzanti/mrmr v0.2.8.

5 SciPy implementation: scipy.stats.ttest_ind of SciPy v1.11.3.

6 SciPy implementation: scipy.stats.shapiro of SciPy v1.11.3.

7 SciPy implementation: scipy.stats.bartlett of SciPy v1.11.3.

8 SciPy implementation: scipy.stats.gaussian_kde of SciPy v1.11.3.
